# Safety and Effectiveness of Bivalirudin in Patients Undergoing Percutaneous Coronary Intervention: A Systematic Review and Meta-Analysis

**DOI:** 10.3389/fphar.2017.00410

**Published:** 2017-07-11

**Authors:** Abdul Hafeez Ahmad Hamdi, Ahmad Fauzi Dali, Thimarul Huda Mat Nuri, Muhammad Syafiq Saleh, Noor Nabila Ajmi, Chin Fen Neoh, Long Chiau Ming, Amir Heberd Abdullah, Tahir Mehmood Khan

**Affiliations:** ^1^Department of Pharmacy Practice, Faculty of Pharmacy, Universiti Teknologi MARA Puncak Alam, Malaysia; ^2^Collaborative Drug Discovery Research Group, Pharmaceutical and Life Sciences CoRe, Universiti Teknologi MARA Shah Alam, Malaysia; ^3^Unit for Medication Outcomes Research and Education, Pharmacy, School of Medicine, University of Tasmania Hobart, TAS, Australia; ^4^School of Pharmacy, KPJ Healthcare University College Nilai, Malaysia; ^5^Vector-Borne Diseases Research Group (VERDI), Pharmaceutical and Life Sciences CoRe, Universiti Teknologi MARA Shah Alam, Malaysia; ^6^Faculty of Health Sciences, Universiti Teknologi MARA Bertam, Malaysia; ^7^School of Pharmacy, Monash University Sunway, Malaysia

**Keywords:** acute coronary syndrome, ischemic events, myocardial infarction, stroke, glycoprotein IIb/IIIa inhibitor, major bleeding, stent thrombosis, percutaneous coronary intervention

## Abstract

Recent clinical trials have shown that while bivalirudin exhibits similar efficacy with heparin, it offers several advantages over heparin, such as a better safety profile. We aimed to evaluate the efficacy and safety of bivalirudin use during Percutaneous Coronary Intervention (PCI) in the treatment of angina and acute coronary syndrome (ACS). We searched the Cochrane Central Register of Controlled Trials (CENTRAL) in the Cochrane Library, PubMed, EMBASE, and Science Direct from January 1980 to January 2016. Randomized controlled trials (RCTs) comparing bivalirudin to heparin during the course of PCI in patients with angina or ACS were included. Outcome measures included all-cause mortality, myocardial infarction, revascularisation, stent thrombosis, stroke, and major bleeding. The selection, quality assessment, and data extraction of the included trials were done independently by four authors, and disagreements were resolved by consensus. Pooled relative risk (RR) estimates and 95% confidence intervals (CIs) were calculated. A total of 12 RCTs involving 44,088 subjects were included. Bivalirudin appeared to be non-superior compared to heparin in reducing all-cause mortality, myocardial infarction, revascularisation, and stroke. Bivalirudin appeared to be related to a higher risk of stent thrombosis when compared to heparin plus provisional use of a glycoprotein IIb/IIIa inhibitor (GPI) at day 30 (RR 1.94 [1.16, 3.24] *p* < 0.01). Overall, bivalirudin-based regimens present a lesser risk of major bleeding (RR 0.56 [0.44–0.71] *p* < 0.001), and Thrombolysis In Myocardial Infarction (TIMI) major bleeding (RR 0.56 [0.43–0.73]) compared with heparin-based regimens either with provisional or routine use of a GPI. However, the magnitude of TIMI major bleeding effect varied greatly (*p* < 0.001), depending on whether a GPI was provisionally used (RR 0.42 [0.34–0.52] *p* < 0.001) or routinely used (RR 0.60 [0.43 –0.83] *p* < 0.001), in the heparin arm. This meta-analysis demonstrated that bivalirudin is associated with a lower risk of major bleeding, but a higher risk of stent thrombosis compared to heparin.

## Introduction

Unstable angina (UA), ST-elevation myocardial infarction (STEMI), and Non-ST-elevation myocardial infarction (NSTEMI) are myocardial ischemic symptoms suggestive of an acute coronary syndrome (ACS). ACS is a term generally used to describe the blockage of blood supply to the heart muscles (Naghavi et al., 2003). Atherosclerotic plaque rupture or erosion, with differing degrees of superimposed thrombosis and distal embolization, results in myocardial under perfusion, which is the basic pathophysiological mechanism of ACS (Rapezzi et al., 2008). Percutaneous coronary intervention (PCI), which works by opening the narrowed arteries thus improving blood flow to the heart, is used to relieve UA and myocardial infarction. This then avoids the need for coronary artery bypass graft (CABG) surgery.

Bivalirudin, a polypeptide, is a direct thrombin inhibitor (DTI) that recognizes thrombin's fibrinogen-binding site, and inhibits the active site of thrombin. Compared to heparin, bivalirudin offers more advantages in that it is less dependent on renal function, and has a lower incidence of anaphylaxis. It is also not inactivated by components of the platelet release reaction (e.g. platelet factor 4), and is able to inhibit clot-bound thrombin. As a result, bivalirudin exhibits less variable levels of anticoagulation compared to heparin (Centurion, 2011). Bivalirudin is indicated for use as an anticoagulant in patients with UA undergoing percutaneous transluminal coronary angioplasty (PTCA). Bivalirudin with provisional use of a glycoprotein IIb/IIIa inhibitor (GPI) is also indicated for use as an anticoagulant in patients undergoing PCI. In patients undergoing PCI, it is indicated for those at risk of having heparin-induced thrombocytopenia (HIT) or heparin-induced thrombocytopenia and thrombosis syndrome (HITTS) (Centurion, 2011).

Meanwhile, heparin has been the mainstay of anticoagulation during PCI in patients with angina and ACS for decades (Jolly and Yusuf, 2010). However, the choice of anticoagulation for PCI remains a hotly-debated issue following the publication of the HEAT PPCI trial (Shahzad et al., 2014) and the BRIGHT trial (Han et al., 2015), both of which favor the use of bivalirudin instead of heparin.

Indeed clinical studies conducted using bivalirudin have demonstrated consistent positive outcomes in patients with stable angina (SA), UA, NSTEMI, and STEMI undergoing PCI. Meta-analyses comparing bivalirudin with unfractionated heparin or enoxaparin plus GPI in patients undergoing PCI showed similar mortality rates and ischemic adverse events, but a reduction in major bleeding complications (De Lucca et al., 2009; Lee et al., 2011). Patient-level analysis using pooled data from the Harmonizing Outcomes with Revascularization and Stents in Acute Myocardial Infarction (HORIZONS-AMI) trial, and the European Ambulance ACS Angiography (EUROMAX) trial revealed that primary PCI with bivalirudin improved net clinical outcomes after 30 days. The pooled analysis also reported that there were significant reductions in major bleeding and thrombocytopenia, but an increase in acute stent thrombosis, compared with heparin ± a GPI (Stone et al., 2015). It must however be noted that most recent data demonstrate that the bleeding benefit identified in previous studies was not due to bivalirudin's properties, but due to a higher incidence of bleeding in the comparator arm due to the disproportionate use of GPIs with heparin (Lee et al., 2011; Mavrakanas and Chatzizisis, 2015; Stone et al., 2015; Fabris et al., 2017). The objective of this systematic review was to thus evaluate the efficacy and safety of bivalirudin use during PCI in the treatment of angina and myocardial infarction. The information on efficacy and safety gained from this systematic review and meta-analysis can be used to assist physicians in making clinical decisions on the choice between bivalirudin and heparin in their daily practice.

## Methods

### Criteria for considering studies for this review

#### Types of studies

Studies were included if they met the following inclusion criteria: randomized controlled trials (RCTs) in which bivalirudin (experimental) was compared with heparin (control), with provisional or routine use of GPIs; and a minimum follow-up period of 30 days from the time of randomization. We excluded studies due to duplication of data, non-RCT design, lack of 30-day event data, bivalirudin being used for an indication other than PCI, or the use of another anticoagulant.

#### Types of participants

Adults aged 18 years or older diagnosed with angina (stable or unstable) or ACS undergoing PCI.

#### Types of interventions

Administration of direct or indirect bivalirudin at any dose compared to heparin, with provisional or routine GPI use.

#### Types of outcome measures

*Efficacy outcomes*

- All-cause mortality at day 30,
- The incidence of myocardial infarction at day 30,
- The need for revascularization at day 30,
- Definite stent thrombosis at day 30,
- Stroke at day 30.

*Safety outcome*

- Major bleeding at day 30.

### Search methods for identification of studies

#### Electronic searches

We searched the Cochrane Central Register of Controlled Trials (Cochrane CENTRAL) in The Cochrane Library, PubMed, EMBASE, and Science Direct for publications dated from January 1980 to January 2016. No language restrictions were imposed. The full search strategies are presented in Supporting Information 1.

### Data collection and analysis

Retrieved citations and full-text papers were examined by four authors independently. Any disagreements were resolved by consensus.

### Data extraction and management

Data from the included studies were extracted by four authors independently using a standardized data extraction form. The form was piloted on four trials to ensure it was suitable for use. Disagreements were resolved by consensus. The extracted information include study characteristics, and patient characteristics. Information related to the outcomes of interest, which included incidence of all-cause mortality, myocardial infarction revascularization, stent thrombosis, stroke, and major bleeding, were extracted from the included studies and entered into Revman Manager Version 5.3 for analysis.

### Assessment of risk of bias and publication bias

Four authors independently examined for publication bias by visual inspection of the funnel plot, and risk of bias of included studies using the Cochrane Collaboration's tool for assessing risk of bias. Disagreements were resolved by consensus. In terms of risk of bias, the following criteria were considered: selection bias (randomization method and allocation concealment); performance bias (blinding of patients and personnel administering the treatment); attrition bias (loss to follow-up); and detection bias (blinding of outcome assessors). The risk of bias in each trial was graded as A: all quality criteria met (low risk of bias); B: one or more criteria partly met (moderate risk of bias); or C: one or more criteria not met (high risk of bias).

### Investigation of heterogeneity and measures of treatment effect

All dichotomous data related to efficacy and safety outcomes were entered into Revman Manager Version 5.3, and analyzed using risk ratio (RR) at a confidence interval (CI) of 95%. Four reviewers independently classified the heterogeneity of the included studies based on statistical and clinical grounds (Yong et al., 2016).

Statistical heterogeneity was examined using the I^2^ statistic (a threshold of 50% was used to define significant heterogeneity), and the Chi^2^ statistic. A fixed-effects model was employed if there was no statistical or clinical heterogeneity. Otherwise, a random-effects model was applied to explore heterogeneity among the included studies.

Subgroup analysis was also performed to assess for factors responsible for heterogeneity among the included studies.

### Quality assessment

Study quality was addressed using the Jadad scale, with scores ranging from 0 to 5. Studies with a score >3 are considered as good quality trials or “good trials,” while studies with a score between 1 and 3 are considered as “poor trials.” The Peter Morris Centre for Evidence in Transplantation^1^.

## Results

### Description of included studies

**Figure 1:**
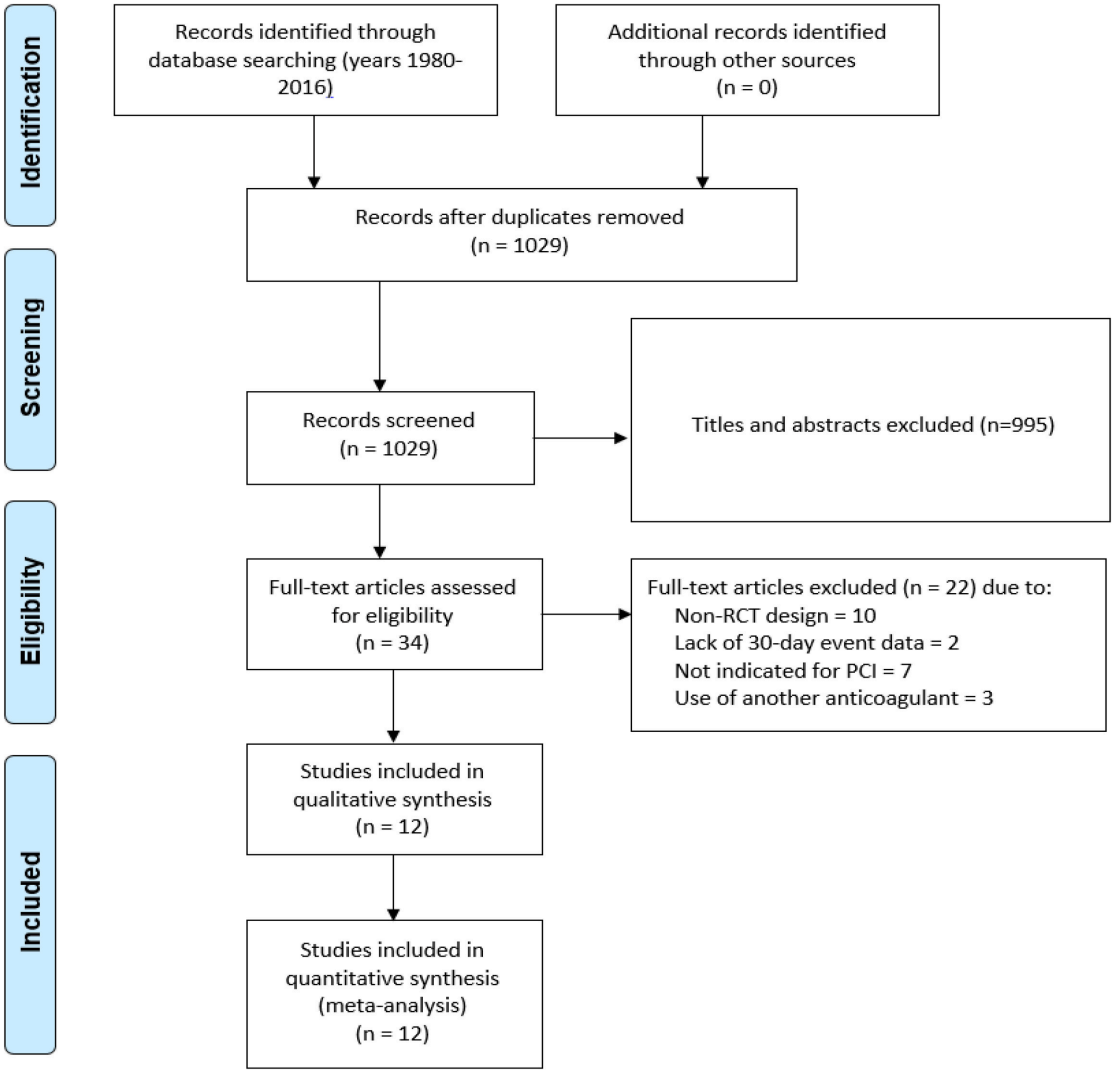
PRISMA diagram demonstrating the search strategy and its results.

We evaluated 1,029 abstracts, of which we assessed 34 full-texts. The search flow diagram is presented in Figure 1. For this systematic review, we included 12 RCTs (Bittl et al., 1995; Lincoff et al., 2003; Stone et al., 2007, 2008; Kastrati et al., 2008, 2011; Tavano et al., 2009; Schulz et al., 2010; Patti et al., 2012; Steg et al., 2013; Shahzad et al., 2014; Han et al., 2015; Valgimigli et al., 2015), which included 44,088 patients that were randomized to either bivalirudin alone (*n* = 21,315), or with heparin with provisional GPI use (*n* = 10,866) (Bittl et al., 1995; Schulz et al., 2010; Patti et al., 2012; Steg et al., 2013; Shahzad et al., 2014; Han et al., 2015), or with heparin with routine GPI use (*n* = 11,722) (Bittl et al., 1995; Lincoff et al., 2003; Stone et al., 2007, 2008; Kastrati et al., 2008, 2011; Tavano et al., 2009; Han et al., 2015) (Supporting Information 2: Characteristics of included studies). Baseline study characteristics are presented in Tables 1, 2, while baseline patient characteristics can be found in Table 3.

**Table 1:** Study characteristics of included trials (Bivalirudin vs. heparin with provisional GPI use).

**Study**	**Reference**	**JADAD score**	**Enrolment period**	**Population**	**Intervention**	**Anticoagulant regimen**	**Patients**	**GPI (%)**
HIRULOG 1995	Bittl et al., 1995	3/5	Mar 1993–July 1994	UA	Bivalirudin alone	Bivalirudin bolus 1.0 mg/kg and infusion 2.5 mg/kg/h during the procedure	2059	0
					UFH alone	UFH 175 IU/kg bolus	2039	0
ISAR-REACT 3 2010	Schulz et al., 2010	5/5	Sept 2005–Jan 2008	UA,SA	Bivalirudin alone	Bivalirudin bolus 0.75 mg/kg and infusion 1.75 mg/kg/h during the procedure	2289	13.5
					UFH alone	UFH 140 IU/kg bolus	2281	15.5
MATRIX 2015	Valgimigli et al., 2015	3/5	Oct 2011–Nov 2014	NSTEMI, STEMI	Bivalirudin alone	Bivalirudin bolus 0.75 mg/kg and infusion 1.75 mg/kg/h during the procedure	3610	5
					UFH + provisional GPI (Abciximab)	UFH 70 IU/kg bolus	3603	7
EUROMAX 2013	Steg et al., 2013	2/5	Mar 2010–June 2013	STEMI	Bivalirudin alone	Bivalirudin bolus 0.75 mg/kg and infusion 1.75 mg/kg/h during the procedure	1089	12
					UFH / LMWH + provisional GPI	UFH 100 IU/kg bolus without a GPI 60 IU/kg with a GPI	1109	69
HEAT-PPCI 2014	Shahzad et al., 2014	4/5	Feb 2012–Nov 2013	STEMI	Bivalirudin alone	Bivalirudin bolus 0.75 mg/kg and infusion 1.75 mg/kg/h during the procedure	905	13
					UFH + provisional GPI	UFH 70 IU/kg bolus	907	15
ARMYDA-7 BIVALVE 2012	Patti et al., 2012	1/5	June 2009–June 2011	UA, NSTEMI, SA	Bivalirudin + provisional GPI	Bivalirudin bolus 0.75 mg/kg and infusion 1.75 mg/kg/h during the procedure	203	12
					UFH + provisional GPI (Abciximab)	UFH 75 IU/kg bolus	198	14
BRIGHT 2015	Han et al., 2015	4/5	Aug 2012–June 2013.	NSTEMI, STEMI	Bivalirudin + provisional GPI	Bivalirudin bolus 0.75 mg/kg and infusion 1.75 mg/kg/h during the procedure	735	4
					UFH + provisional GPI	UFH 100 IU/kg bolus	729	6

*GPI, Glycoprotein IIb/IIIa inhibitor, UA, unstable angina, SA, stable angina, NSTEMI, non-st-elevation myocardial infarction, STEMI, ST-elevation myocardial infarction, MI, myocardial infarction, UFH, Unfractionated heparin*.

**Table 2:** Study characteristics of included trials (Bivalirudin vs. heparin with routine GPI use).

**Study**	**Reference**	**JADAD score**	**Enrolment period**	**Population**	**Intervention**	**Anticoagulant regimen**	**Patients**	**GPI (%)**
NAPLES 2009	Tavano et al., 2009	2/5	Oct 2005–Feb 2008	UA,SA	Bivalirudin alone	Bivalirudin bolus 0.75 mg/kg and infusion 1.75 mg/kg/h during the procedure	167	1
					UFH + routine GPI (Tirofiban)	UFH 70 IU/kg bolus	168	100
REPLACE-2 2003	Lincoff et al., 2003	4/5	Oct 2001–Aug 2002	UA,SA,MI	Bivalirudin + provisional GPI (Abciximab)	Bivalirudin bolus 0.75 mg/kg and infusion 1.75 mg/kg/h during the procedure	2994	7
					UFH + routine GPI (Abciximab)	UFH 65 IU/kg bolus	3008	97
ACUITY 2006	Stone et al., 2006	2/5	Aug 2003–Dec 2005	UA,NSTEMI	Bivalirudin alone	Bivalirudin bolus 0.1 mg/kg bolus and infusion 0.25 mg/kg/h during procedure	4612	9
					Bivalirudin + routine GPI	Biivalirudin bolus 0.5 mg/kg bolus and infusion 1.75 mg/kg/h during procedure	4604	97
					UFH or Enoxaparin + routine GPI	UFH 60 IU/kg bolus and 12 IU/kg/h infusion. Enoxaparin 1 mg/kg Q12H SQ with additional 0.3 mg/kg or 0.75 mg/kg bolus IV before PCI if SQ dose more than 8 or 16 h, respectively	4603	97
ISAR-REACT 4 2011	Kastrati et al., 2011	5/5	Sept 2005–Jan 2008	NSTEMI	Bivalirudin alone	Bivalirudin bolus 0.75 mg/kg and infusion 1.75 mg/kg/h during the procedure	860	0
					UFH + routine GPI (Abciximab)	UFH 70 IU/kg bolus	861	100
BRIGHT 2015	Han et al., 2015	4/5	Aug 2012–June 2013.	NSTEMI, STEMI	Bivalirudin + provisional GPI	Bivalirudin bolus 0.75 mg/kg and infusion 1.75 mg/kg/h during the procedure	735	4
					UFH + routine GPI (Tirofiban)	UFH 60 IU/kg bolus	730	100
HORIZON-AMI 2008	Stone et al., 2008	4/5	Mar 2005–May 2007	STEMI	Bivalirudin alone	Bivalirudin bolus 0.75 mg/kg and infusion 1.75 mg/kg/h during the procedure	1800	8
					UFH + routine GPI	Bivalirudin bolus 0.75 mg/kg and infusion 1.75 mg/kg/h during the procedure	1802	98

*GPI: Glycoprotein IIb/IIIa inhibitor, UA: unstable angina, SA: stable angina, NSTEMI: non-st-elevation myocardial infarction, STEMI: ST-elevation myocardial infarction, MI: myocardial infarction, UFH: Unfractionated heparin*.

**Table 3:** Patients' characteristics of included studies.

**Study**	**Intervention**	**Mean age (s.d)**	**Male n (%)**	**Female n (%)**	**Hypertension n (%)**	**Diabetes Melitus n (%)**	**Hyperlipidemia n (%)**	**Smoking n (%)**	**Prior STEMI n (%)**	**Prior AMI n (%)**	**Prior SA n (%)**	**Prior UA n (%)**	**Prior NSTEMI n (%)**	**Prior CABG n (%)**	**Prior PCI n (%)**	**Prior Stroke n (%)**
HIRULOG 1995	Bivalirudin alone	63	NA	670 (33)	NA	1440 (21)	NA	NA	NA	NA	NA	1707 (83)	NA	NA	NA	NA
	UFH alone	62	NA	652 (32)	NA	419 (21)	NA	NA	NA	NA	NA	1687 (83)	NA	NA	NA	NA
ISAR-REACT 3 2010	Bivalirudin alone	66.9 (10)	NA	545 (23.8)	2034 (88.9)	618 (27.0)	1850 (80.8)	328 (14.3)	NA	734 (32.1)	NA	NA	NA	286 (12.5)	NA	NA
	UFH alone	67.0 (10)	NA	530 (23.2)	2044 (89.6)	636 (27.9)	1795 (78.7)	337 (14.8)	NA	689 (30.2)	NA	NA	NA	248 (10.9)	NA	NA
MATRIX 2015	Bivalirudin alone	65.4 (11.9)	2731 (75.7)	879 (24.3)	2264 (62.7)	815 (22.6)	1596 (44.2)	2020 (56.0)	NA	530 (14.7)	NA	NA	NA	127 (3.5)	536 (14.8)	181 (5.0)
	UFH + provisional GPI (Abciximab)	65.4 (11.9)	2764 (76.7)	839 (23.3)	2222 (61.7)	786 (21.8)	1558 (43.2)	2016 (56.0)	NA	500 (13.9)	NA	NA	NA	95 (2.6)	504 (14.0)	185 (5.1)
EUROMAX 2013	Bivalirudin alone	275 (25.3)	459 (42.2)	127 (11.7)	398 (36.6)	453 (41.6)	NA	80 (7.4)	NA	NA	NA	18 (1.7)	97 (8.9)	NA	NA	NA
	UFH / LMWH + provisional GPI	248 (22.4)	504 (45.5)	169 (15.3)	417 (37.6)	472 (42.6)	NA	113 (10.2)	NA	NA	NA	29 (2.6)	108 (9.7)	NA	NA	NA
HEAT-PPCI 2014	Bivalirudin alone	63	NA	285 (29)	362 (40)	362 (40)	327 (37)	371 (42)	NA	122 (14)	NA	NA	NA	22 (2)	76 (8)	NA
	UFH + provisional GPI		NA	244 (27)	388 (43)	388 (43)	342 (38)	379 (43)	NA	93 (10)	NA	NA	NA	20 (2)	54 (6)	NA
ARMYDA-7 BIVALVE 2012	Bivalirudin + provisional GPI	70.3 (8.4)	141 (71)	NA	178 (89)	134 (67)	175 (88)	35 (17)	NA	74 (37)	137 (69)	38 (19)	NA	NA	NA	NA
	UFH + provisional GPI (Abciximab)	70.1 (9.7)	148 (72)	NA	187 (92)	120 (59)	172 (84)	27 (13)	NA	69 (34)	149 (73)	30 (14)	NA	NA	NA	NA
BRIGHT 2015	Bivalirudin + provisional GPI	57.3 (11.6)	608 (82.7)	NA	301 (41.0)	168 (22.9)	266 (36.5)	463 (63.0)	NA	32 (4.4)	NA	NA	NA	NA	37 (5.0)	63 (8.6)
	UFH + provisional GPI	58.1 (11.7)	595 (81.6)	NA	312 (42.8)	137 (18.8)	275 (38.0)	429 (58.8)	NA	33 (4.5)	NA	NA	NA	NA	35 (4.8)	63 (8.6)
	UFH + routine GPI (Tirofiban)	58.2 (11.8)	599 (82.1)	NA	311 (42.6)	160 (21.9)	267 (36.8)	449 (61.5)	NA	33 (4.5)	NA	NA	NA	NA	37 (5.1)	53 (7.3)
NAPLES 2009	Bivalirudin alone	65.0 (9.8)	110 (65.9)	57 (34.1)	125 (74.9)	167 (100)	105 (62.9)	34 (20.4)	NA	75 (44.9)	NA	NA	NA	12 (7.2)	46 (27.5)	NA
	UFH + routine GPI (Tirofiban)	65.6 (8.3)	108 (64.3)	60 (35.7)	131 (78.0)	168 (100)	109 (64.9)	35 (20.8)	NA	75 (44.6)	NA	NA	NA	15 (8.9)	41 (24.4)	NA
REPLACE-2 2003	Bivalirudin + provisional GPI	62.6 (10.8)	NA	758 (25.3)	1965 (66.0)	840 (28.1)	NA	796 (27.2)	NA	1099 (37.4)	NA	NA	NA	538 (18.0)	1029 (34.5)	75 (2.5)
	UFH + routine GPI (Abciximab)	62.6 (11.0)	NA	779 (25.9)	2040 (68.0)	784 (26.1)	NA	762 (26.0)	NA	1085 (36.7)	NA	NA	NA	564 (18.8)	1059 (35.3)	66 (2.2)
ACUITY 2006	Bivalirudin alone	3	3195 (69.3)	NA	3080 (67.1)	1287 (28.1)	2579 (57.0)	1312 (29.0)	NA	1431 (31.8)	NA	NA	NA	818 (18.9)	1820 (39.9)	NA
	Bivalirudin + routine GPI		3216 (69.9)	NA	3074 (67.2)	1267 (27.7)	2588 (57.4)	1323 (29.3)	NA	1372 (30.5)	NA	NA	NA	801 (19.1)	1720 (37.8)	NA
	UFH or enoxaparin + routine GPI		3249 (70.6)	NA	3058/4577 (66.8)	1298/4564 (28.4)	2580/4511 (57.2)	1308/4508 (29.0)	NA	1419/4493 (31.6)	NA	NA	NA	834/4588 (18.2)	1780/4567 (39.0)	NA
ISAR-REACT 4 2011	Bivalirudin alone	67.5 (10.8)	NA	199 (23.1)	727 (84.5)	243 (28.3)	580 (67.4)	195 (22.7)	NA	163 (19.0)	NA	NA	NA	89 (10.3)	267 (31.0)	NA
	UFH + routine GPI (Abciximab)	67.5 (11.2)	NA	200 (23.2)	745 (86.5)	257 (29.8)	600 (69.7)	215 (25.0)	NA	188 (21.8)	NA	NA	NA	92 (10.7)	292 (33.9)	NA
HORIZON-AMI 2008	Bivalirudin alone	59.8	1388 (77.1)	412 (22.9)	931 (51.8)	281 (15.6)	781 (43.4)	845 (47.2)	NA	187 (10.4)	NA	NA	NA	59 (3.3)	188 (10.5)	NA
	UFH + routine GPI	60.7	1372 (76.1)	430 (23.9)	993 (55.2)	312 (17.3)	769 (42.7)	807 (45.0)	NA	205 (11.4)	NA	NA	NA	46 (2.6)	198 (11.0)	NA

*GPI, glycoprotein IIb/IIIa inhibitor, UA, unstable angina, SA, stable angina, NSTEMI, non-st-elevation myocardial infarction, STEMI, ST-elevation myocardial infarction, MI, myocardial infarction, UFH, Unfractionated heparin, PCI, percutaneous coronary intervention, CABG, coronary artery bypass graft surgery*.

### Risk of bias of included studies

#### Allocation

**Figure 2:**
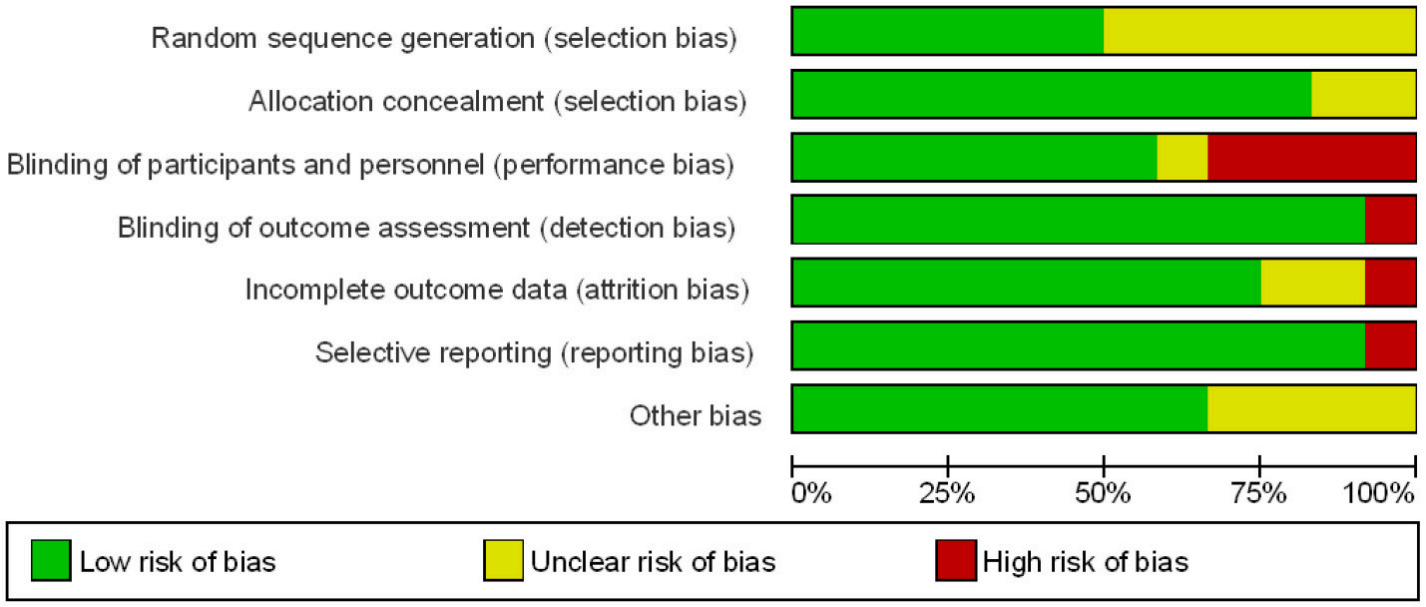
Methodological quality graph: review authors' judgements about each methodological quality item presented as percentages across all included studies.

**Figure 3:**
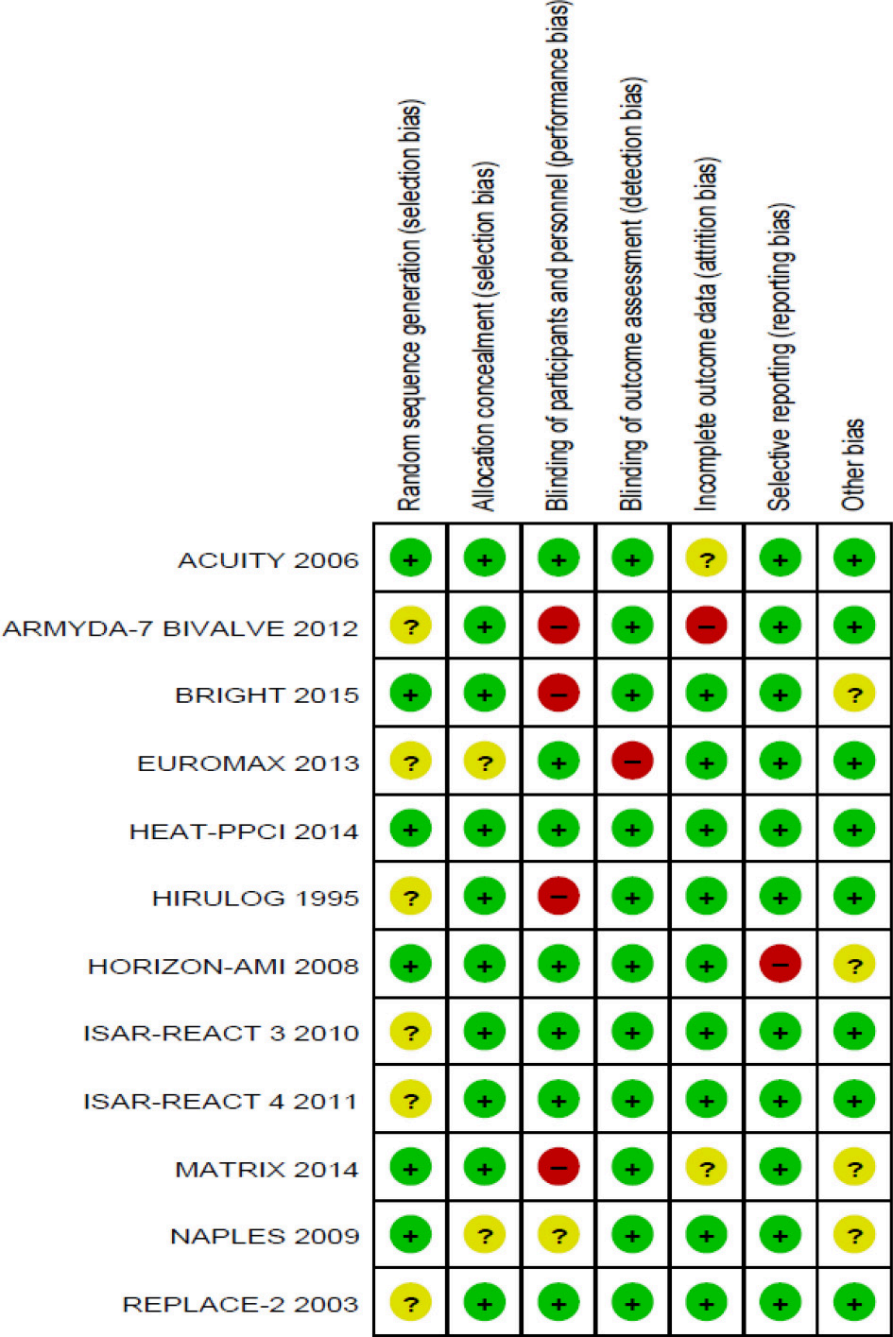
Methodological quality summary: review authors' judgements about each methodological quality item for each included study.

Six of the twelve trials reported an appropriate method of randomization through the use of a central computerized system for random sequence generation. In the remaining six trials, the methods used for random sequence generation were not described in detail. However, appropriate allocation concealment through a central telephonic or web-based voice system was reported in 10 out of the 12 trials evaluated. The methods of allocation concealment were not reported in detail in the other two trials (Figures 2, 3).

### Blinding

Blinding of participants and personnel were observed in seven studies (double blind). Four studies were not blinded (open-label), and blinding in one trial was not stated clearly. However, blinding of outcome assessments was observed in the majority of studies (11 out of 12), where independent clinical committees who were unaware of treatment assignments were responsible in the adjudication of the clinical outcomes, hence reducing detection bias in these studies. The blinding of outcome assessments was not performed in one particular trial (Figures 2, 3).

### Incomplete outcome data

Nine studies reported all incomplete outcome data, along with justifications that include patient withdrawal, loss to follow-up, and patients not receiving trial medications. Two studies were observed to have missing outcome data. Details are shown in Figures 2, 3.

### Other potential sources of bias

The funnel plots showed asymmetry in all the outcomes of interest in this study (Supporting Information 3). This reflects fundamental methodological heterogeneity, such as different clinical conditions and treatment strategies used, but it is not likely that the asymmetry was due to publication bias.

All the included studies were either funded by for-profit organizations, or the authors received research support or consultant fees from the pharmaceutical companies that are associated with the study drug. However, the studies were conducted, and the data analyzed in an independent fashion by steering or safety committees and/or the research institutes in charge. There was no clear rationale to suggest biased reporting based on this link between the authors and the companies that funded the research.

### Efficacy and safety outcomes

Outcomes evaluating the clinical efficacy and safety of bivalirudin compared to heparin with provisional or routine GPI use were assessed as below.

### All-cause mortality

**Figure 4:**
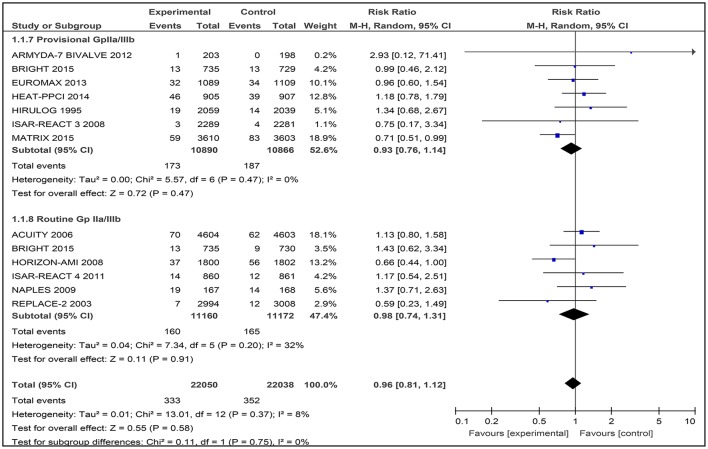
Forest plot comparing 30-day all-cause mortality in patients randomized to bivalirudin vs. heparin with provisional or routine GPI use.

Twelve studies contributed to the overall analysis of mortality, with 44,088 patients included (Figure 4). Among the 12 included studies, there was no difference between bivalirudin vs. heparin for all-cause mortality (RR 0.96 [0.81–1.12], I^2^ 8%).

#### Bivalirudin vs. heparin with provisional GPI use

The incidence of 30-day all cause death did not differ significantly in the bivalirudin arm compared with the heparin plus provisionally administered GPI arm: 173 of 10,890 patients (1.59%) receiving bivalirudin died, compared with 187 of 10,866 patients receiving heparin plus GPI (1.72%) (RR 0.93 [0.76–1.14], I^2^ 0%).

#### Bivalirudin vs. heparin with routine GPI use

No substantial difference in 30-day mortality emerged between the two strategies: 160 of 11,160 patients (1.43%) receiving bivalirudin compared with 165 of 11,172 patients (1.48%) receiving heparin, died (RR 0.98 [0.74 – 1.31], I^2^ 32%).

### Myocardial infarction

All 12 studies reported on the incidence of myocardial infarction at day 30, with a total of 44,088 patients with angina or ACS included. No difference in the occurrence of myocardial infarction between bivalirudin vs. heparin was observed (RR 1.04 [0.93–1.16], I^2^ 34%) [Supporting Information 4(1)].

A further subgroup analysis conducted revealed that heterogeneity was significantly higher when comparing between bivalirudin vs. heparin plus provisional GPI use, which was due to the HEAT-PPCI trial (Shahzad et al., 2014). However, no significant difference in the occurrence of myocardial infarction was reported (RR 1.01 [0.87–1.42], I^2^ 63%). Hence, this study was excluded in the subgroup analysis for provisional GPI use as it was not combinable with other studies.

#### Bivalirudin vs. heparin with provisional GPI use

**Figure 5:**
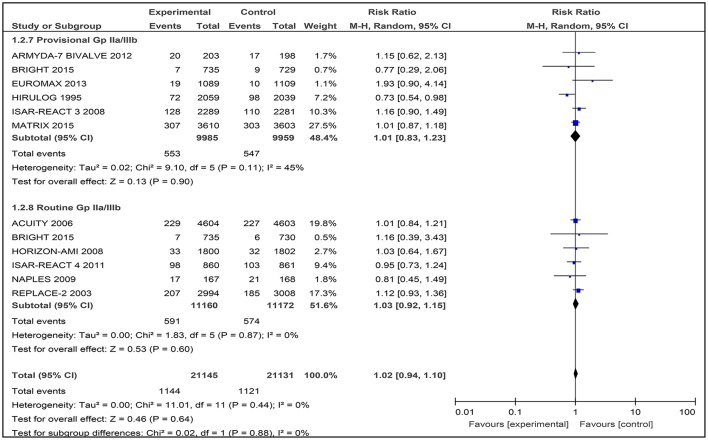
Forest plot comparing 30-day myocardial infarction in patients randomized to bivalirudin vs. heparin with provisional or routine GPI use.

Among the six studies comparing bivalirudin vs. heparin with provisional GPI use, there was no significant difference in the occurrence of myocardial infarction observed, reported in 553 of 9,985 patients (5.54%) receiving bivalirudin compared with 574 of 9,959 patients (5.76%) receiving heparin (RR 1.01 [0.83–1.23], I^2^ 45%) (Figure 5).

#### Bivalirudin vs. heparin with routine GPI use

Among the six studies comparing bivalirudin vs. heparin with routine GPI use, there was no significant difference in the occurrence of myocardial infarction observed, reported in 591 of 11,160 patients (5.30%) receiving bivalirudin compared with 574 of 11,172 patients (5.14%) receiving heparin (RR 1.03 [0.92–1.15], I^2^ 0%) (Figure 5).

### Revascularization

All 12 included studies reported on the need for revascularization procedures at 30 days. However, in the first analysis conducted, heterogeneity was significantly higher in the overall analysis as well as in the subgroup analysis involving provisional use of a GPI. There were significant differences in the outcomes observed where a higher incidence of revascularization was reported in the overall comparison (RR 1.50 [1.09–2.06], I^2^ 74%) [Supporting Information 4(2)] as well as in the provisional use of a GPI (RR 1.81 [1.10–2.97], I^2^ 75%) [Supporting Information 4(2)]. The main source of this heterogeneity was the HIRULOG trial (Bittl et al., 1995) as it has the largest weight. Therefore, this trial was excluded.

**Figure 6:**
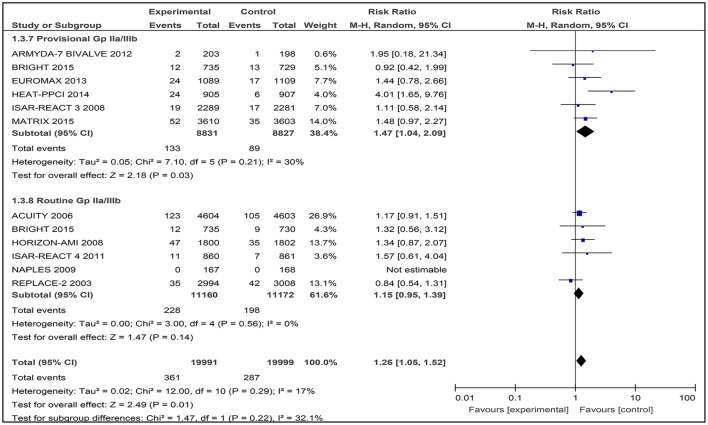
Forest plot comparing 30-day revascularization in patients randomized to bivalirudin vs. heparin with provisional or routine GPI use.

Eleven studies were then used in the meta-analysis, involving a total of 39,990 patients with angina or ACS. There was a statistically significant difference observed in this outcome where a higher number of patients who received bivalirudin (361 of 19,991 patients; 1.81%) needed to undergo revascularization procedures compared to those receiving heparin (287 of 19,999 patients; 1.44%) (RR 1.26 [1.05–1.52], I^2^ 17%) (Figure 6).

#### Bivalirudin vs. heparin with provisional GPI use

In this subgroup analysis that included data from six trials, the incidence of revascularisation was also reported to be significantly higher in the bivalirudin group (133 of 8,831 patients; 1.51%) compared to the heparin plus provisional GPI use arm (89 of 8,827 patients; 1.00%) (RR 1.47 [1.04–2.09], I^2^ 30%) (Figure 6).

#### Bivalirudin vs. heparin with routine GPI use

Among the six studies comparing bivalirudin vs. heparin with routine GPI use, there was no significant difference in the rate of revascularization observed: 228 of 11,160 patients (2.04%) receiving bivalirudin compared with 198 of 11,172 patients (1.77%) receiving heparin, underwent revascularization (RR 1.15 [0.95–1.39], I^2^ 0%) (Figure 6). No event of revascularization was reported in both the bivalirudin and heparin group in the NAPLES trial.

### Stent thrombosis

**Figure 7:**
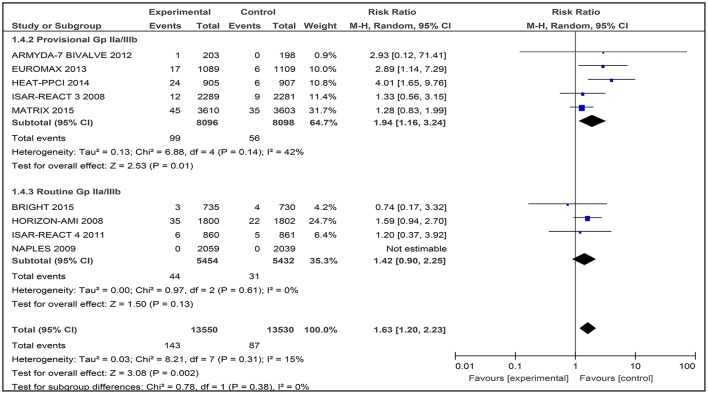
Forest plot comparing definite stent thrombosis events in patients randomized to bivalirudin vs. heparin with provisional or routine GPI use.

A total of nine studies involving 27,080 patients was assessed for the number of thrombosis events after treatment with bivalirudin compared with heparin with provisional GPI or with routine GPI (Figure 7).

#### Bivalirudin vs. heparin with provisional GPI use

For comparison between the bivalirudin group vs. the heparin with provisional GPI use group, there was moderate heterogeneity (*I*^2^ = 42%) that was statistically significant (RR 1.94 [1.16, 3.24]) (Figure 7). Thus, the use of heparin with provisional GPI use may reduce the number of definite stent thrombosis events in these patients.

#### Bivalirudin vs. heparin with routine GPI use

For bivalirudin vs. heparin with routine use of a GPI, the RR was observed to be RR 1.42 [CI 95% 0.90, 2.25, I^2^ 0%]. However, for the NAPLES trial, due to the lack of data, no estimates were generated which might have influenced the overall effect (Figure 7).

### Stroke

**Figure 8:**
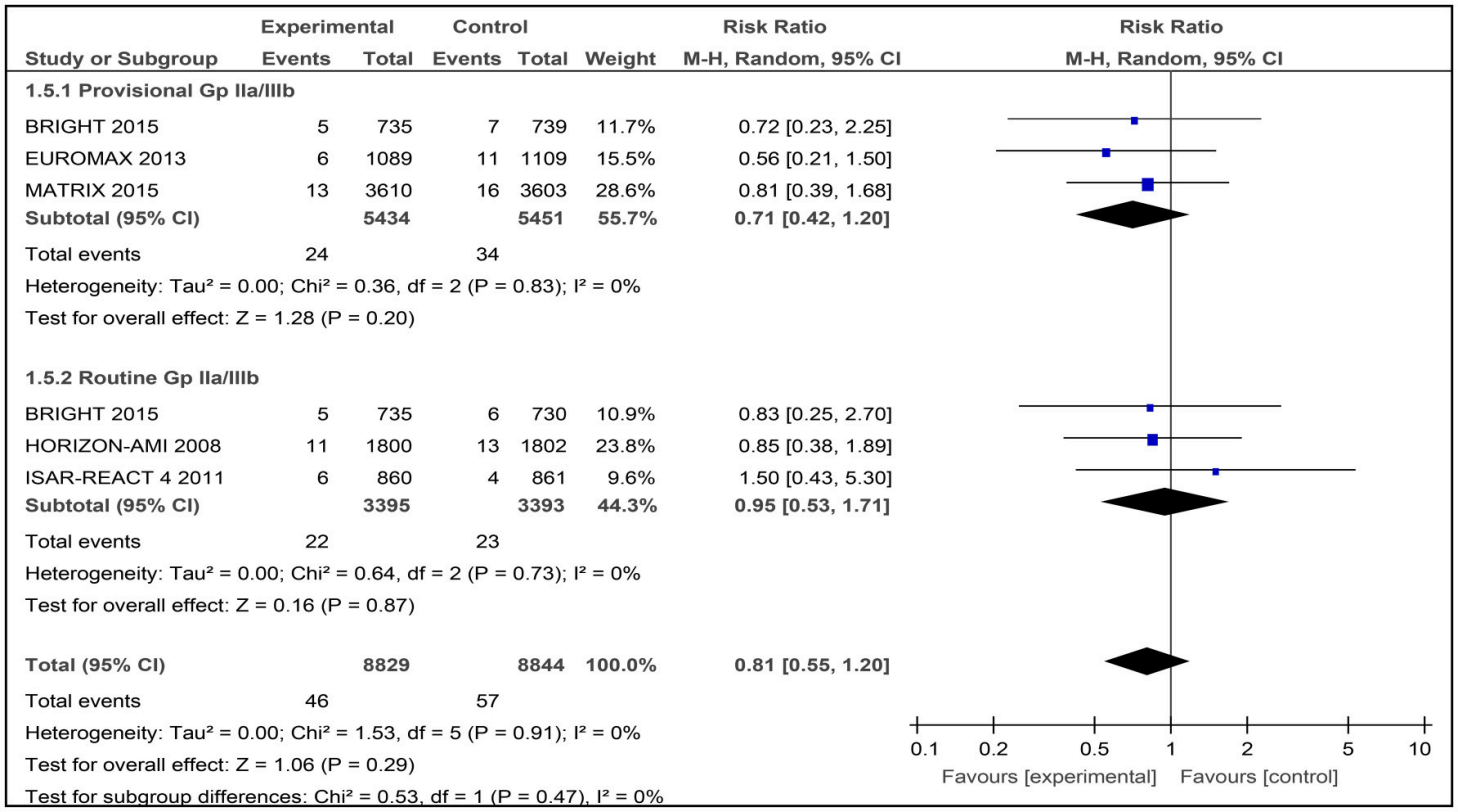
Forest plot comparing stroke events in patients randomized to bivalirudin vs. heparin with provisional or routine GPI use.

Five studies were assessed for the number of stroke events after patients had been given bivalirudin together with heparin plus provisional GPI use and/or heparin plus routine GPI use. A total of 17,673 patients were involved. In the BRIGHT trial, both the use of heparin with provisional and routine use of a GPI were tested (Figure 8).

#### Bivalirudin vs. heparin with provisional GPI use

For bivalirudin vs. heparin with provisional use of a GPI, the RR was 0.71 [0.42, 1.20] with an I^2^ 0%.) However, the overall effect was not statistically significant (*Z* = 1.28, *p* = 0.20; Figure 8).

#### Bivalirudin vs. heparin with routine GPI use

For the use of bivalirudin vs. heparin with routine GPI use, the RR was 0.95 [0.53, 1.71], and the overall effect was not statistically significant (Figure 8).

### Safety outcomes

#### Major bleeding

The primary definition of major bleeding at 30-day follow-up varied between the included trials (Supporting Information 5). This includes definitions based on either the REPLACE trial (Lincoff et al., 2003), ACUITY trial (Stone et al., 2006), the Bleeding Academic Research Consortium (BARC) definition, or the Thrombolysis in Myocardial Infarction (TIMI) definition (Mehran et al., 2011).

**Figure 9:**
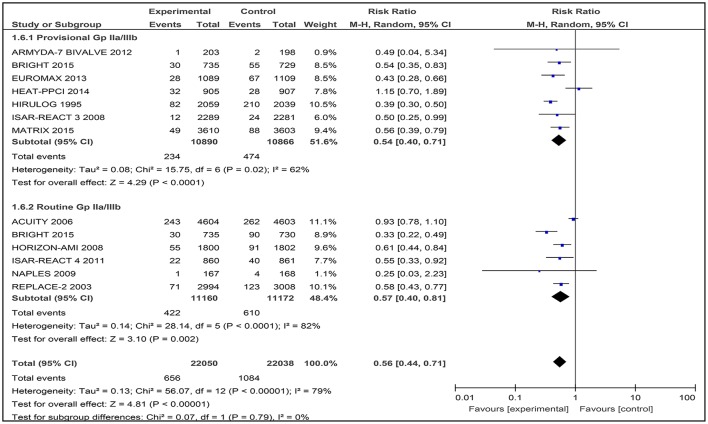
Forest plot comparing major bleeding utilizing the study definitions in patients randomized to bivalirudin vs. heparin with provisional or routine GPI use.

Major bleeding was assessed in all the included studies, which involved 44,088 patients. In the pooled analyses, 1740 (3.95%) patients had a major bleeding. There was significantly less major bleeding with bivalirudin compare to heparin with provisional or routine GPI use (RR 0.56, [0.44–0.71]). However, there was significant heterogeneity between the trials (*I*^2^ = 79%; Figure 9).

The results of bivalirudin vs. heparin with provisional or routine GPI use are presented as a narrative synthesis, and the summary of the safety outcome on major bleeding at 30-days based on study definitions is presented in Table 4.

**Table 4:** Summary of trials comparing 30-day major bleeding events based on the primary study definition in patients randomized to bivalirudin vs. heparin with provisional and routine GPI use.

**Study**	**Reference**	**Primary major bleeding definition**	***N***	**Indication**	**RR [95% CI]: *p*-value**
**BIVALIRUDIN VS. HEPARIN + PROVISIONAL GPI**					
ARMYDA-7 BIVALVE 2012	Patti et al., 2012	TIMI	401	SA, UA, NSTEMI	0.49 [0.04, 5.34]: 0.0001
BRIGHT 2015	Han et al., 2015	BARC	1464	NSTEMI, STEMI	0.54 [0.35, 0.83]: < 0.001
EUROMAX 2013	Steg et al., 2013	ACUITY trial	2198	STEMI	0.43 [0.28, 0.66]: < 0.001
HEAT-PPCI 2014	Shahzad et al., 2014	BARC	1812	STEMI	1.15 [0.70, 1.89]: 0.59
HIRULOG 1995	Bittl et al., 1995	TIMI	4098	UA, Post-infarction Angina	0.39 [0.30, 0.50]: 0.001
ISAR-REACT 3 2008	Kastrati et al., 2008	REPLACE 2 trial	4570	UA, SA	0.50 [0.25, 0.99]: 0.008
MATRIX 2015	Valgimigli et al., 2015	BARC	7213	NSTEMI, STEMI	0.56 [0.39, 0.79]: < 0.001
**BIVALIRUDIN VS. HEPARIN + ROUTINE GPI**					
ACUITY 2006	Stone et al., 2006	ACUITY trial	9207	UA, NSTEMI	0.93 [0.78, 1.10]: < 0.001
BRIGHT 2015	Han et al., 2015	BARC	1465	NSTEMI, STEMI	0.33 [0.22, 0.49]: < 0.001
HORIZON-AMI 2008	Stone et al., 2008	ACUITY trial	3602	STEMI	0.61 [0.44, 0.84]: < 0.001
ISAR-REACT 4 2011	Kastrati et al., 2011	REPLACE 2 trial	1721	NSTEMI	0.55 [0.33, 0.92]: 0.02
NAPLES 2009	Tavano et al., 2009	ACUITY trial	335	SA, UA	0.25 [0.03, 2.23]: 0.371
REPLACE-2 2003	Lincoff et al., 2003	REPLACE 2 trial	6002	SA, UA, MI	0.58 [0.43, 0.77]: 0.001

*TIMI, Thrombolysis in Myocardial Infarction; BARC, Bleeding Academic Research Consortium; REPLACE-2, Randomized Evaluation in PCI Linking Angiomax to Reduced Clinical Events; ACUITY, Acute Catheterization and Urgent Intervention Triage Strategy*.

#### Bivalirudin vs. heparin with provisional GPI use

Analysis of seven included trials comparing bivalirudin vs. heparin with provisional GPI use, found that bivalirudin use was associated with a significant decrease in major bleeding incidence (study definition) across six included trials. HEAT-PPCI 2014 (Shahzad et al., 2014) was the only trial that reported a non-significant difference in the rate of major bleeding between bivalirudin and heparin with provisional GPI use (3.5 vs. 3.1%; RR 1.15 [0.70 to 1.89]) (Table 4).

#### Bivalirudin vs. heparin with routine GPI use

Among the six studies comparing bivalirudin vs. heparin with routine GPI use, there was a significant difference in the rate of major bleeding across five studies: treatment with bivalirudin, as compared with heparin with routine GPI use, was associated with a lower incidence of major hemorrhage. However, in all six studies except the NAPLES trial, the rate of major bleeding in both groups was not significant (0.6 vs. 2.4%; RR 0.25 [0.03 to 2.23]).

#### Major bleeding (TIMI classification)

The TIMI bleeding criteria has been used for the past 30 years, and has been reported in most cardiovascular trials (Mehran et al., 2011). Subgroup analysis was conducted across studies that provided major bleeding data based on the TIMI definition.

**Figure 10:**
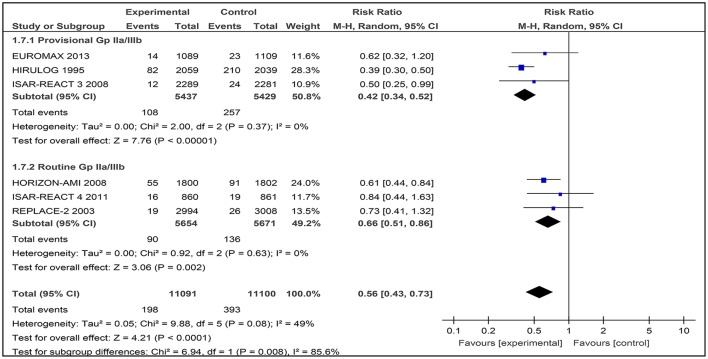
Forest plot comparing major bleeding (TIMI definition) in patients randomized to bivalirudin vs. heparin with provisional or routine GPI use.

Generally, the use of bivalirudin was associated with a significant decrease in TIMI major bleeding rates across studies with routine or provisional use of a GPI (RR 0.56 [0.43–0.73]), with moderate heterogeneity observed between the trials (I^2^ 49%; Figure 10).

#### Bivalirudin vs. heparin with provisional GPI use

Among the three included studies, 108 out of 5,437 patients (1.98%) received bivalirudin while 257 out of 5,429 (4.73%) received heparin with provisional GPI use. There was a significant difference in the occurrence of TIMI major bleeding where the rate of major bleeding was lower in the bivalirudin group than in the heparin with provisional GPI use (RR 0.42 [0.34–0.52]) group. Low heterogeneity was observed between the trials (I^2^ = 0%).

#### Bivalirudin vs. heparin with routine GPI use

TIMI major bleeding occurred in 90 out of 5,654 patients (1.59%) who received bivalirudin and 136 out of 5,671 patients (2.40%) who received heparin with routine GPI use. The incidence of TIMI major bleeding differed significantly between these two groups (RR 0.66 [0.51–0.86] I^2^ = 0%).

Overall, bivalirudin-based regimens lowered the risk of TIMI major bleeding, but the magnitude of this effect varied depending on whether GPIs were provisionally (RR 0.42 [0.34–0.52]), or routinely (RR 0.66 [0.51–0.86]) used in the heparin arm.

## Discussion

### Efficacy and safety outcomes

#### All-cause mortality

The findings of this systematic review and meta-analysis comparing anticoagulation with bivalirudin vs. heparin with or without a GPI for PCI suggest no difference in death at 30-day follow-up (1.51% in bivalirudin group; 1.60% in heparin group). Our analysis was consistent with previous meta-analyses which demonstrated that bivalirudin was not superior compared to heparin with or without a GPI in reducing death in patients with angina or ACS undergoing PCI (Cavender and Sabatine, 2014; Cassese et al., 2015; Navarese et al., 2015).

The use of bivalirudin or heparin in patients undergoing PCI has been a long-debated issue, with conflicting evidence reported in different RCTs. Despite the latest trials, such as MATRIX (Valgimigli et al., 2015) and BRIGHT (Han et al., 2015) that showed an apparent benefit in all-cause mortality where death was significantly lower in the bivalirudin group compared to those receiving heparin, previous RCTs have showed no difference in this primary outcome except for the HORIZON-AMI trial (Stone et al., 2008). In this updated meta-analysis, consistent with other previous systematic reviews and meta-analyses, the results of these two latest trials have not shown many differences in overall mortality. Thus, this concludes that bivalirudin is not superior compared to heparin with provisional or routine GPI use in reducing death at 30 days. However, bivalirudin may be advantageous in reducing death in patients undergoing PCI who are having NSTEMI or STEMI, as these are the populations included in the three trials namely, MATRIX, BRIGHT, and HORIZON-AMI; which have shown positive outcomes in terms of all-cause mortality when patients were given bivalirudin instead of heparin (Stone et al., 2008; Han et al., 2015; Valgimigli et al., 2015).

#### Myocardial infarction

In the analysis for incidence of myocardial infarction following the administration of anticoagulants, it was found that no statistically significant difference was observed between bivalirudin and heparin with or without routine GPI use at 30 days follow-up (5.30% in bivalirudin group; 5.14% in heparin group). Our analysis is consistent with earlier meta-analyses which indicated that bivalirudin was similar to heparin with or without routine use of a GPI in reducing reinfarction rates in patients with angina or ACS undergoing PCI recently (Cavender and Sabatine, 2014; Cassese et al., 2015; Navarese et al., 2015).

The first subgroup analysis conducted for provisional and routine use of a GPI also displayed similar results to the overall risk of developing myocardial infarction. However, heterogeneity was found to be high in the subgroup with provisional GPI use (I^2^ = 63%), which was also consistent with a recent meta-analysis reported by Navarese et al. (2015). The main source of heterogeneity was the HEAT-PPCI trial (Shahzad et al., 2014), which showed a 3-fold incidence (24 cases in bivalirudin groups and 8 cases in heparin groups) of myocardial infarction in the bivalirudin group compared to the heparin group. This difference was thought to be due to the way the HEAT-PPCI trial was conducted, in which a delayed consent approach was used in order to recruit a higher proportion of potentially eligible participants and avoid selective inclusion of participants (Shahzad et al., 2014; Mavrakanas and Chatzizisis, 2015). However, after excluding the HEAT-PPCI trial in another subgroup analysis (Shahzad et al., 2014), and including only 6 studies that were combinable: ARMYDA-7 BIVALVE (Patti et al., 2012), BRIGHT (Han et al., 2015), EUROMAX (Steg et al., 2013), HIRULOG (Bittl et al., 1995), ISAR-REACT 3 (Schulz et al., 2010), and MATRIX (Valgimigli et al., 2015); a similar non-significant incidence of myocardial infarction was also observed.

In the other subgroup of routine use of a GPI, similar insignificant results were observed. This was mainly due to the six included trials that reported neither bivalirudin nor heparin to be more superior in attenuating the risk of myocardial infarction post PCI.

#### Revascularisation

The forest plot of studies with bivalirudin vs. heparin with both routine and provisional use of a GPI demonstrated homogeneity of datasets, with *I*^2^ = 0% and *I*^2^ = 30%, respectively. However, when we included the HIRULOG trial in the forest plot, heterogeneity was observed in the incidence of revascularization at day-30 with provisional GPI use, but not with routine GPI use, *I*^2^ = 75% [see Supporting Information 4(2)].

Our findings are similar with other meta-analyses with bivalirudin vs. heparin with routine GPI use (Bittl et al., 1995; Lincoff et al., 2003; Stone et al., 2006; Steg et al., 2013).

Overall, the HEAT-PPCI trial had a RR of 4.01 [1.65, 9.76], indicating a four-time greater incidence of revascularization compared to other studies. In addition, the HEAT-PPCI (Shahzad et al., 2014) trial was the only trial that showed a significant difference in the rate of revascularization after PCI, with 24 cases out of 905 patients in the bivalirudin group (2.65%) vs. six cases out of 907 patients (0.66%) in the heparin group (RR 4.01 [1.65, 9.76] *p* < 0.001).

#### Stent thrombosis

For the outcome of definite stent thrombosis, a total of nine studies was analyzed, out of which four studies involved the use of heparin with routine GPI. The meta-analysis consisting of the BRIGHT trial, HORIZON-AMI trial, ISAR-REACT 4 trial, and the NAPLES trial were not statistically significant. However, in the HORIZON-AMI trial, there was an increased risk of acute stent thrombosis within 24 h in the bivalirudin group, but no significant increase was observed at day-30 as mentioned earlier.

#### Stroke

Of the five included studies that tested bivalirudin vs. heparin with provisional GPI or routine GPI use, no statistically significant results were found for reduction of stroke events after conducting PCI.

The use of heparin with provisional GPI use did not show any difference at day-30 in the BRIGHT trial, EUROMAX trial, and MATRIX trial. In these studies, events of stroke were quantitatively small compared to studies with larger sample sizes. This resulted in statistically insignificant results when the pooled results were analyzed.

The use of heparin with routine GPI use after 30 days did no reveal any difference in the reduction of stroke events in the BRIGHT trial, HORIZON-AMI trial, and ISAR-REACT 4 trial. In fact, all studies were statistically insignificant. Thus, the use of heparin with provisional and routine GPI use does not show any superiority to bivalirudin alone in reducing the number of stroke events after PCI.

### Safety outcome

#### Major bleeding

In this meta-analysis, bivalirudin-based regimens reduced the risk of major bleeding compared with heparin-based regimens either with provisional or routine use of a GPI, although the definitions for major bleeding (Supporting Information 5) and indications for PCI varied; which contributed to high heterogeneity among the trials (*I*^2^ = 79%).

These results were comparable with a number of meta-analyses conducted recently (Cavender and Sabatine, 2014; Cassese et al., 2015; Nairooz et al., 2015). However, in another meta-analysis conducted by Navarese et al. (2015), no significant difference was found in the risk for major bleeding (based on the primary study definition) between bivalirudin-based regimens and heparin-based regimens with provisional GPI use (Navarese et al., 2015). This difference was mainly due to the exclusion of trials, such as MATRIX, HIRULOG, and ISAR-REACT 3 (Bittl et al., 1995; Schulz et al., 2010; Valgimigli et al., 2015).

In further analyses, bivalirudin-based regimens significantly lowered the risk of TIMI major bleeding, with consistent homogeneity between the trials. However, the magnitude of reduction varied depending on whether GPIs were provisionally or routinely used in a heparin-based regimen. This finding was similar to a meta-analysis conducted by Dong et al. (2013) where bivalirudin-based regimens significantly lowered the risk of TIMI major bleeding, and the incidence of major bleeding was higher in patients assigned to heparin plus routine GPI use (Dong et al., 2013).

As shown in Table 1, the percentage of GPI use in bivalirudin and heparin-based regimens varied between all trials. A meta-analysis conducted by Nairooz et al. (2015) suggested that the lower risk of major bleeding offered by bivalirudin-based regimens may be driven by mandating the use of a GPI (Nairooz et al., 2015). The improved safety of bivalirudin-based regimens could be largely attributed to the more frequent co-administration of GPIs with heparin. However, when the rate of GPI use was balanced between bivalirudin-based regimens and heparin-based regimens, there was no difference in the incidence of major bleeding (Huang et al., 2015).

In this scenario, the effects of GPI use should not be underestimated when comparing different anticoagulants during PCI (Huang et al., 2015). Routine GPI use in addition to pre-treatment with loading doses of clopidogrel, have significantly increased the risk of bleeding complications due to the excessively enhanced anti-platelet effect (Dong et al., 2013). Dong et al. (2013) evaluated the relative safety and efficacy of upstream vs. delayed administration of the GPI in STEMI patients, and observed no beneficial effects in terms of 30-day clinical outcomes. Thus, the authors did not advocate for routine utilization of upstream GPI in STEMI patients treated with primary PCI (Dong et al., 2013). Generally, the routine use of a GPI before PCI does not seem to be as beneficial as was previously thought (Bagai et al., 2013). Further clinical trials are needed to verify the association with major bleeding between routine vs. provisional GPI use with a balanced usage rate between bivalirudin-based regimens and heparin-based regimens to find therapies that could improve reperfusion with minimum bleeding risk.

Based on the current consensus, clinicians should begin reporting bleeding events according to the BARC definition. The standardization of definitions for bleeding (Supporting Information 5) will overcome the limitations of the use of non-standardized definitions of major bleeding between studies, and will allow for a more consistent reporting of bleeding in future clinical investigations (Steg et al., 2011).

#### Quality assessment of the evidence

Potential sources of bias reported in the included studies are: did not perform or disclose details of randomization and allocation concealment (Bittl et al., 1995; Lincoff et al., 2003; Kastrati et al., 2008; Tavano et al., 2009; Schulz et al., 2010; Patti et al., 2012; Steg et al., 2013), and lack of blinding of participants (Bittl et al., 1995; Tavano et al., 2009; Patti et al., 2012; Han et al., 2015; Valgimigli et al., 2015; Figure 3). Nevertheless, these factors do not adversely affect the validity of the findings as blinding of the outcome assessments were ensured throughout the course of most of the trials. Besides that, the authors of the four open-label trials justified that compliance to study protocol was ensured to minimize the effect of non-blinding (Bittl et al., 1995; Patti et al., 2012; Han et al., 2015; Valgimigli et al., 2015). Apart from that, three trials have incomplete outcome results (Stone et al., 2006; Patti et al., 2012; Valgimigli et al., 2015).

Thus, for future studies, the use of modern computerized and telephone systems to ensure adequate randomization and allocation, imposing double-blinding methods, and ensuring proper follow-up periods and documentation; may enhance and strengthen the conclusions that can be drawn from the findings.

The presence of heterogeneity in some of the outcome analyses could be explained by the diverse methodological designs of the included studies, which may be attributed to the inclusion of both angina (SA, UA) and ACS (NSTEMI, STEMI) in the analysis, as well as different co-morbidities in the included participants.

It was also noted that some of the studies were either sponsored by pharmaceutical companies associated with the study drug, or the authors of the included studies received research support or consultant fees from the relevant pharmaceutical companies. However, there was no strong evidence to suggest the results published could be compromised.

## Limitations

The first limitation is that this systematic review includes all patients referred for PCI (SA, UA, STEMI, and NSTEMI). This may introduce heterogeneity as we included and analyzed patients from the different extremes of STEMI to those with SA. Another limitation is that we only included studies with 30-day event data, and did not include long-term follow-up data. In addition to that, non-standardized definitions of major bleeding for the different studies used in this meta-analysis were also a setback in being able to draw sound conclusions on the safety of bivalirudin compared to heparin. Future efforts should thus be made to report standard measures of major bleeding based on the BARC or TIMI definitions (Mehran et al., 2011). Finally, though random effects pooling reduces heterogeneity, heterogeneity was still observed among the included studies. This necessitated the need to exclude certain studies to be able to form combinable groups for analysis of myocardial and revascularisation outcomes.

## Conclusions

This meta-analysis of 12 RCTs involving 44,088 patients demonstrated that bivalirudin is associated with a lower risk of major bleeding, but a higher risk of stent thrombosis compare to heparin-based therapies. Bivalirudin appeared to be non-superior compared to heparin in reducing all-cause mortality, myocardial infarction, revascularisation, and stroke.

## Author contributions

AHAH, AFD, THM, MSS, NNA, CFN, LCM, AHA, and TMK contributed to the database search, data collection, data extraction, data analysis, and writing of the manuscript. The topic was conceptualized by TMK.

### Conflict of interest statement

The authors declare that the research was conducted in the absence of any commercial or financial relationships that could be construed as a potential conflict of interest.

## Footnotes

^1^The Peter Morris Centre for Evidence in Transplantation. *The Transplant Library-Assessment of Rcts*. Available online at: http://www.transplantevidence.com/library-rct.php (Accessed February 4, 2016).
