# Supplementary material for: Safety and Effectiveness of Bivalirudin in Patients Undergoing Percutaneous Coronary Intervention: A Systematic Review and Meta-Analysis
Source: Front Pharmacol. 2017 Jul 11;8:410. doi: 10.3389/fphar.2017.00410 (PMC5504279; doi:10.3389/fphar.2017.00410)
Supplement: Supplementary file 1 [file DataSheet1.DOCX]

### Supporting Information 1: Search strategies used

**CENTRAL**

#1 Mesh descriptor Myocardial Ischemia explode all trees

#2 Myocardial Ischemi*

#3 heart muscle ischemi*

#4 angina

#5 Mesh descriptor myocardial infarct* explode all trees

#6 heart infarct*

#7 acute coronar*

#8 coronary syndrome*

#9 Preinfarct*

#10 Pre Infarct*

#11 STEMI

#12 NONSTEMI

#13 NON-STEMI

#14 NSTEMI

#15 ACS

#16 Mesh descriptor Acute Coronary Syndrome explode all trees

#17 Mesh descriptor Stable Angina explode all trees

#18 (#1 OR #2 OR #3 OR #4 OR #5 OR #6 OR #7 OR #8 OR #9 OR #10 OR #11 OR #12 OR #13 OR #14 OR #15 OR #16 OR #17)

#19 Bivalirudin

#20 Hirulog

#21 Hirulog-1

#22 Angiomax

#23 Mesh descriptor Antithrombins explode all trees

#24 (#19 OR #20 OR #21 OR #22 OR #23)

#25Mesh descriptor Heparin explode all trees

#26 Mesh descriptor unfractionated heparin explode all trees

#27 UFH

#28Mesh descriptor Low Molecular Weight Heparin explode all trees

#29Mesh descriptor Enoxaparin explode all trees

#30MeshdescriptorNadroparin explode all trees

#31Mesh descriptor Dalteparin explode all trees

#32Tinzaparin

#33 (#25 OR #26 OR #27OR #28 OR #29 OR #30OR #31 OR #32)

#34 (#18 AND #24 AND #33)

**PubMed**

#1 Myocardial Ischemia [Mesh]

#2 Myocardial Ischemi*

#3 heart muscle ischemi*

#4 angina

#5 myocardial infarct*

#6 heart infarct*

#7 acute coronar*

#8 coronary syndrome*

#9 Preinfarct*

#10 Pre Infarct*

#11 STEMI

#12 NONSTEMI

#13 NON-STEMI

#14 NSTEMI

#15 ACS

#16 Acute Coronary Syndrome [Mesh]

#17 Stable Angina [Mesh]

#18 (#1 OR #2 OR #3 OR #4 OR #5 OR #6 OR #7 OR #8 OR #9 OR #10 OR #11 OR #12 OR #13 OR #14 OR #15 OR #16 OR #17)

#19 Bivalirudin

#20 Hirulog

#21 Hirulog-1

#22 Angiomax

#23 (#19 OR #20 OR #21 OR #22)

#24 Antithrombin [Mesh]

#25 Heparin [Mesh]

#26 unfractionated heparin

#27 UFH

#28 Low Molecular Weight Heparin [Mesh]

#29 Enoxaparin

#30 Nadroparin

#31 Dalteparin

#32 Tinzaparin

#33 (#24 OR #25 OR #26 OR #27 OR #28 OR #29OR #30 OR #31 OR #32)

#34 (#18 AND #23 AND #33)

**EMBASE**

#1 Myocardial Ischemi*

#2heart muscle ischemi*

#3 angina

#4 myocardial infarct*

#5 heart infarct*

#6 acute coronar*

#7 coronary syndrome*

#8Preinfarct*

#9 Pre Infarct*

#10 STEMI

#11 NONSTEMI

#12 NON-STEMI

#13 NSTEMI

#14 ACS

#15 'Acute Coronary Syndrome'/exp

#16 'Heart Infarction'/exp

#17 'Heart Muscle Ischemia'/exp

#18 'non ST segment elevation myocardial infarction'/exp

#19 'ST segment elevation myocardial infarction'/exp

#20 'unstable angina pectoris'/exp

#21 'angina pectoris'/exp

#22 'stable angina pectoris'/exp

#23 (#1 OR #2 OR #3 OR #4 OR #5 OR #6 OR #7 OR #8 OR #9 OR #10 OR #11 OR #12 OR #13 OR #14 OR #15 OR #16#17#18#19#20#21#22)

#24 Bivalirudin

#25 'hirulog'/exp

#26 Hirulog-1

#27 Angiomax

#28 (#25 OR #26 OR #27 OR #28)

#29 'heparin'/exp

#30 unfractionated heparin

#31 UFH

#32 'low molecular weight heparin'/exp

#33 Enoxaparin

#34 Nadroparin

#35 Dalteparin

#36 Tinzaparin

#37'antithrombin'/exp

#38 (#30 OR #31 OR #32 OR #33 OR #34 OR #35 OR #36 OR #37#38)

#39 (#24 AND #29 AND #38) – FINAL

**Science Direct**

(Myocardial Ischemi* OR heart muscle ischemi* OR angina OR myocardial infarct* OR heart infarct* OR acute coronar* OR coronary syndrome* OR Preinfarct* OR STEMI OR NONSTEMI OR NON-STEMI OR NSTEMI OR ACS OR Acute Coronary Syndrome OR Stable Angina

(Bivalirudin OR Hirulog OR Hirulog-1 OR Angiomax) AND (Heparin OR unfractionated heparin OR UFH OR Low Molecular Weight Heparin OR Enoxaparin OR Nadroparin OR Dalteparin OR Tinzaparin OR Antithrombin)
