# Supplementary material for: Safety and Effectiveness of Bivalirudin in Patients Undergoing Percutaneous Coronary Intervention: A Systematic Review and Meta-Analysis
Source: Front Pharmacol. 2017 Jul 11;8:410. doi: 10.3389/fphar.2017.00410 (PMC5504279; doi:10.3389/fphar.2017.00410)
Supplement: Supplementary file 2 [file DataSheet2.docx]

# Supporting Information 2: Characteristics of included studies

### ACUITY 2006

| Enrollment period | Aug 2003– Jan 2007 | |
| --- | --- | --- |
| Study design | Multicenter,open label, randomized trial. | |
| Study aim | To compared heparin plus a GPI, bivalirudin plus a GPI,and bivalirudin alone in patients with moderate or high-risk acute coronary syndromes who were undergoing an early invasive strategy. | |
| Outcome | Bivalirudin plus a GPI, as compared with heparin plus a GPI, was associated with non inferior 30-day rates of the composite ischemia end point (7.7% and 7.3%, respectively), Major bleeding (5.3% and 5.7%), and the net clinical outcome end point (11.8% and 11.7%).  Bivalirudin alone, as compared with heparin plus a GPI, was associated with a non inferior rate of the composite ischemia end point (7.8% and 7.3%, respectively; P = 0.32; relative risk, 1.08; 95% confidence interval [CI], 0.93 to 1.24) and significantly reduced rates of major bleeding (3.0% vs. 5.7%; p< 0.001; relative risk, 0.53; 95% CI, 0.43 to 0.65) and the net clinical outcome end point (10.1% vs. 11.7%; P = 0.02; relative risk, 0.86; 95% CI, 0.77 to 0.97). | |
| Risk of bias | | |
| **Item** | **Author’s Judgement** | **Description** |
| Random sequence generation  (selection bias) | Low risk | The allocation sequence generation was defined clearly.  Quote: "randomization was performed in blocks of six, stratified according to the site and the use of or intent to administer a thienopyridine before angiography".  Comment: Probably done |
| Allocation concealment (selection bias) | Low Risk | Allocation was adequately concealed.  Quote: "Telephone randomization performed".  Comment: Probably done |
| Blinding of participants and personnel (performance bias) | Low risk | No blinding, but the review authors judge that the outcome and the outcome measurement are not likely to be influenced by lack of blinding.  Quote: "Several limitations of the present study deserve comment. First, the logistic complexities of the trial necessitated an open-label design, which introduced the potential for bias. However, the frequency of the provisional use of GPIs for procedural complications related to PCI among patients in the group receiving bivalirudin monotherapy was nearly identical to that in the double-blind REPLACE-2 trial,9 and all endpoint events were adjudicated by an independent committee that was unaware of the treatment assignments"  Comment: Probably done |
| Blinding of outcome assessment  (detection bias) | Low risk | The outcome assessment was blinded.  Quote: “A clinical events committee that was unaware of the treatment assignments adjudicated all primary end-point events with the use of original source documents”.  Comment: Probably done |
| Incomplete outcome data (attrition bias) | Unclear risk | Insufficient reporting of attrition/exclusions to permit judgement of ‘Yes’ or ‘No’.  Comment: There was missing data while conducting the trials. No reasons for missing data provided. |
| Selective reporting  (reporting bias) | Low risk | The reports of the study was free of suggestion of selective outcome reporting.  Comment: The study protocol is available and all of the study’s pre-specified (primary and secondary) outcomes that are of interest in the review have been reported in the pre-specified way. |
| Other bias | Low Risk | The study appears to be free of other sources of bias. |

**Jadad Score**

| NO | ITEM | SCORE |
| --- | --- | --- |
| 1 | Was the study described as randomized (this includes words such as randomly, random, and randomization)? | 1 |
| 2 | Was the method used to generate the sequence of randomization described and appropriate (table of random numbers, computer generated, etc.? | 1 |
| 3 | Was the study described as double blind? | 0 |
| 4 | Was the method of double blinding described and appropriate (identical placebo, active placebo, dummy, etc.? | 0 |
| 5 | Was there a description of withdrawals and dropouts? | 0 |
| 6 | Deduct one point if the method used to generate the sequence of randomization was described and it was inappropriate (patients were allocated alternately, or according to date of birth, hospital number, etc.). | 0 |
| 7 | Deduct one point if the study was described as double blind but the method of blinding was inappropriate (e.g., comparison of tablet vs. injection with no double dummy). | 0 |

*Jadad scores range from 0 to 5. Trial scoring 3 or greater is considered as good quality trial (‘good trials’ = Jadad score of 4 or 5) vs. ‘poor trials’ = Jadad score of 1-3)*

For this Acuity study total jaded score is 2/5, so we can consider this study is a poor trial, and this trial is clearly stated open labeled.

### ARMYDA-7 BIVALVE 2012

| Enrollment period | June 2009– June 2011 | |
| --- | --- | --- |
| Study design | Multicenter, open label, randomized trial | |
| Study aim | To evaluate the safety and efficacy of bivalirudin versus UFH in selected PCI patients presenting with 1 of the following features of elevated bleeding risk: age 75 years, diabetes mellitus, and chronic renal failure. | |
| Outcome | Occurrence of the primary safety end point was 1.5% in the bivalirudin group and 9.9% in the UFH group (p 0.0001); this benefit was essentially driven by the prevention of entry-site hematomas >10 cm (0.5% vs 6.9%, p 0.002). | |
| Risk of bias | | |
| **Item** | **Author’s Judgement** | **Description** |
| Random sequence generation  (selection bias) | Unclear Risk | Insufficient information to permit judgement of ‘Yes’ or ‘No’.  Comment: The allocation sequence generation was not defined clearly. |
| Allocation concealment (selection bias) | Low risk | Allocation was adequately concealed.  Quote: "Eligible patients were assigned to the allocation arm using an electronic spreadsheet indicating group assignment".  Comment: Probably done |
| Blinding of participants and personnel (performance bias) | High risk | No blinding.  Quote: " Study drug administration was performed on an open-label basis without blinding"  Comment: No justification from author whether this will affect the outcome. |
| Blinding of outcome assessment  (detection bias) | Low risk | The outcome assessment was blinded.  Quote: “Physicians performing laboratory testing as well as those adjudicating events during hospitalization were not aware of the randomization assignment; follow-up evaluation was obtained by office visit at 1 month, when hemoglobin levels were also measured and when a blinded investigator adjudicated events occurring from discharge to 30 days”.  Comment: Probably done |
| Incomplete outcome data (attrition bias) | High Risk | Missing outcome data was not mentioned in the study. |
| Selective reporting  (reporting bias) | Low risk | The reports of the study was free of suggestion of selective outcome reporting.  Comment: The study protocol is available and all of the study’s pre-specified (primary and secondary) outcomes that are of interest in the review have been reported in the pre-specified way. |
| Other bias | Low Risk | The study appears to be free of other sources of bias. |

**Jadad score**

| NO | ITEM | SCORE |
| --- | --- | --- |
| 1 | Was the study described as randomized (this includes words such as randomly, random, and randomization)? | 1 |
| 2 | Was the method used to generate the sequence of randomization described and appropriate (table of random numbers, computer generated, etc.? | 0 |
| 3 | Was the study described as double blind? | 0 |
| 4 | Was the method of double blinding described and appropriate (identical placebo, active placebo, dummy, etc.? | 0 |
| 5 | Was there a description of withdrawals and dropouts? | 0 |
| 6 | Deduct one point if the method used to generate the sequence of randomization was described and it was inappropriate (patients were allocated alternately, or according to date of birth, hospital number, etc.). | 0 |
| 7 | Deduct one point if the study was described as double blind but the method of blinding was inappropriate (e.g., comparison of tablet vs. injection with no double dummy). | 0 |

*Jadad scores range from 0 to 5. Trial scoring 3 or greater is considered as good quality trial (‘good trials’ = Jadad score of 4 or 5) vs. ‘poor trials’ = Jadad score of 1-3)*

Scoring for the Jadad score is 1/5 (poor quality) for this trial, the allocation sequence generation was not defined clearly, and study drug administration was performed on an open-label basis without blinding"

### BRIGHT 2014

| Enrollment period | August 2012 - June 2013. | |
| --- | --- | --- |
| Study design | Multicenter, open-label , randomized trial | |
| Study aim | To determine if bivalirudin is superior to heparin alone and to heparin plus tirofiban during primary PCI. | |
| Outcome | Among patients with AMI undergoing primary PCI, the use of bivalirudin with a median 3-hour post procedure PCI-dose infusion resulted in a decrease in net adverse clinical events compared with both heparin alone and heparin plus tirofiban. This finding was primarily due to a reduction in bleeding events with bivalirudin, without significant differences in major adverse cardiac or cerebral events or stent thrombosis | |
| **Item** | **Author’s Judgement** | **Description** |
| Random sequence generation  (selection bias) | Low Risk | The allocation sequence generation was defined clearly.  Quote: "Patients were randomly assigned without stratification by STEMI vs NSTEMI to receive bivalirudin alone, heparin alone, or heparin plus tirofiban in a 1:1:1 ratio using sealed envelopes with a block size of 6".  Comment: Probably done |
| Allocation concealment (selection bias) | Low Risk | Allocation was adequately concealed.  Quote: "Patients were randomly assigned without stratification by STEMI vs NSTEMI to receive bivalirudin alone, heparin alone, or heparin plus tirofiban in a 1:1:1 ratio using sealed envelopes with a block size of 6".  Comment: Probably done |
| Blinding of participants and personnel (performance bias) | High risk | No blinding.  Quote: " an investigator sponsored, large-scale, multicenter, randomized, open-label study designed"  Comment: No justification from author whether this will affect the outcome. |
| Blinding of outcome assessment  (detection bias) | Low Risk | The outcome assessment was blinded.  Quote: “All net adverse clinical events and stent thrombosis events were adjudicated by an independent clinical events committee blinded to randomization assignment”.  Comment: Probably done |
| Incomplete outcome data (attrition bias) | Low Risk | Incomplete outcome data were adequately addressed.  Quote: “All analyses are by intention to treat…at 30 days, 7 patients excluded (2 withdrew consent, 5 lost to follow-up”  Comment: Probably done |
| Selective reporting  (reporting bias) | Low Risk | The reports of the study was free of suggestion of selective outcome reporting.  Comment: The study protocol is available and all of the study’s pre-specified (primary and secondary) outcomes that are of interest in the review have been reported in the pre-specified way. |
| Other bias | Unclear Risk | Insufficient information to assess whether an important risk of bias exists.  Comment: The study was sponsored by the company of the intervention drug. |

| NO | ITEM | SCORE |
| --- | --- | --- |
| 1 | Was the study described as randomized (this includes words such as randomly, random, and randomization)? | 1 |
| 2 | Was the method used to generate the sequence of randomization described and appropriate (table of random numbers, computer generated, etc.? | 1 |
| 3 | Was the study described as double blind? | 0 |
| 4 | Was the method of double blinding described and appropriate (identical placebo, active placebo, dummy, etc.? |  |
| 5 | Was there a description of withdrawals and dropouts? | 1 |
| 6 | Deduct one point if the method used to generate the sequence of randomization was described and it was inappropriate (patients were allocated alternately, or according to date of birth, hospital number, etc.). | 1 |
| 7 | Deduct one point if the study was described as double blind but the method of blinding was inappropriate (e.g., comparison of tablet vs. injection with no double dummy). | 0 |

**Jadad Score**

*Jadad scores range from 0 to 5. Trial scoring 3 or greater is considered as good quality trial (‘good trials’ = Jadad score of 4 or 5) vs. ‘poor trials’ = Jadad score of 1-3)*

Jaded score for this study is 4/5 , considering this is good quality trial.

### EUROMAX 2013

| Enrollment period | Mar 2010– June 2013 | |
| --- | --- | --- |
| Study design | Multicenter, open-label , randomized trial | |
| Study aim | To compare antithrombotic therapy with bivalirudin or unfractionated heparin during emergency transport for primary percutaneous coronary intervention (PPCI) | |
| Outcome | Bivalirudin, started during transport for primary PCI, improved 30-day clinical outcomes with a reduction in major bleeding but with an increase in acute stent thrombosis. | |
| Risk of bias | | |
| **Item** | **Author’s Judgement** | **Description** |
| Random sequence generation  (selection bias) | Unclear risk | Insufficient information to permit judgement of ‘Yes’ or ‘No’.  Comment: The allocation sequence generation was not defined clearly. |
| Allocation concealment (selection bias) | Unclear Risk | Insufficient information to permit judgement of ‘Yes’ or ‘No’.  Comment: Method of concealment is not described to allow for definite judgement. |
| Blinding of participants and personnel (performance bias) | Low Risk | No blinding, but the review authors judge that the outcome and the outcome measurement are not likely to be influenced by lack of blinding.  Quote: An open-label design was implemented because of the logistic impracticality of a double-blind design in the context of emergency transport for primary PCI in a multinational trial. To minimize reporting bias that can be associated with an open-label design, events were adjudicated blindly by an independent clinical-events committee that used standardized end-point definitions.,"  Comment: Probably done |
| Blinding of outcome assessment  (detection bias) | High risk | The outcome assessment was blinded.  Quote: “An independent clinical-events committee whose members were unaware of study-group assignments adjudicated deaths, bleeding episodes, reinfarction, ischemia-driven revascularization, stent thrombosis, and stroke”.  Comment: Probably done |
| Incomplete outcome data (attrition bias) | Low risk | Incomplete outcome data were adequately addressed.  Comment: “Data on cardiac-related history were missing for one patient in each study group”.  Comment: Probably done |
| Selective reporting  (reporting bias) | Low risk | The reports of the study was free of suggestion of selective outcome reporting.  Comment: The study protocol is available and all of the study’s pre-specified (primary and secondary) outcomes that are of interest in the review have been reported in the pre-specified way. |
| Other bias | Low Risk | The study appears to be free of other sources of bias. |

**Jadad score**

| NO | ITEM | SCORE |
| --- | --- | --- |
| 1 | Was the study described as randomized (this includes words such as randomly, random, and randomization)? | 1 |
| 2 | Was the method used to generate the sequence of randomization described and appropriate (table of random numbers, computer generated, etc.? | 0 |
| 3 | Was the study described as double blind? | 0 |
| 4 | Was the method of double blinding described and appropriate (identical placebo, active placebo, dummy, etc.? | 0 |
| 5 | Was there a description of withdrawals and dropouts? | 1 |
| 6 | Deduct one point if the method used to generate the sequence of randomization was described and it was inappropriate (patients were allocated alternately, or according to date of birth, hospital number, etc.). | 0 |
| 7 | Deduct one point if the study was described as double blind but the method of blinding was inappropriate (e.g., comparison of tablet vs. injection with no double dummy). | 0 |

*Jadad scores range from 0 to 5. Trial scoring 3 or greater is considered as good quality trial (‘good trials’ = Jadad score of 4 or 5) vs. ‘poor trials’ = Jadad score of 1-3*

This study can be considered poor quality, Jadad score is 2/5.

### HEAT-PPCI 2014

| Enrollment period | Feb 2012– Nov 2013 | |
| --- | --- | --- |
| Study design | Single center, open-label, randomized trial | |
| Study aim | To compare antithrombotic therapy with bivalirudin or unfractionated heparin during primary percutaneous coronary intervention (PPCI) | |
| Outcome | Compared with bivalirudin, heparin reduces the incidence of major adverse ischaemic events in the setting of PPCI, with no increase in bleeding complications. suggests a significant advantage in favour of heparin over bivalirudin, mainly in terms of a reduced incidence of acute stent thrombosis and associated recurrent myocardial infarction. | |
| Risk of bias | | |
| **Item** | **Author’s Judgement** | **Description** |
| Random sequence generation  (selection bias) | Low risk | The allocation sequence generation was defined clearly.  Quote: "We randomly allocated participants (1:1) at presentation to the Liverpool Heart and Chest Hospital and before entry to the cardiac catheterization laboratory, by use of a dedicated computer running a bespoke randomization application as its sole software function".  Comment: Probably done |
| Allocation concealment (selection bias) | Low risk | Allocation was adequately concealed.  Quote: "using sequential use of serial numbered, opaque envelopes, which is separated into four distinct groups reflecting the stratification options".  Comment: Probably done |
| Blinding of participants and personnel (performance bias) | Low Risk | No blinding, but the review authors judge that the outcome and the outcome measurement are not likely to be influenced by lack of blinding.  Quote: "We used an open-label design but the outcome measures were overt clinical events, supported by objective clinical observations, even in the case of reinfarction. All events were subject to masked assessment. An open-label design was also used in the HORIZONS-AMI and EUROMAX trials”  Comment: Probably done |
| Blinding of outcome assessment  (detection bias) | Low risk | The outcome assessment was blinded.  Quote: “All primary efficacy and safety outcome measures and stent thrombosis events were assessed by an independent Clinical Events Committee. The members of this committee were masked to treatment group allocation”.  Comment: Probably done |
| Incomplete outcome data (attrition bias) | Low risk | Incomplete outcome data were adequately addressed.  Quote: “17 patients died before the investigators were able to obtain consent (7 heparin group, 10 bivalirudin group). Consent was not available for a further 17 surviving patients (13 contact lost before consent, 1 withdrawal and three refusals). These patients have been excluded from the analyses but investigators know, from national mortality tracking, that all were alive at 28 days after their randomization.”  Comment: Probably done |
| Selective reporting  (reporting bias) | Low risk | The reports of the study was free of suggestion of selective outcome reporting.  Comment: The study protocol is available and all of the study’s pre-specified (primary and secondary) outcomes that are of interest in the review have been reported in the pre-specified way. |
| Other bias | Low Risk | The study appears to be free of other sources of bias. |

**Jadad score**

| NO | ITEM | SCORE |
| --- | --- | --- |
| 1 | Was the study described as randomized (this includes words such as randomly, random, and randomization)? | 1 |
| 2 | Was the method used to generate the sequence of randomization described and appropriate (table of random numbers, computer generated, etc.? | 1 |
| 3 | Was the study described as double blind? | 0 |
| 4 | Was the method of double blinding described and appropriate (identical placebo, active placebo, dummy, etc.? | 0 |
| 5 | Was there a description of withdrawals and dropouts? | 1 |
| 6 | Deduct one point if the method used to generate the sequence of randomization was described and it was inappropriate (patients were allocated alternately, or according to date of birth, hospital number, etc.). | 1 |
| 7 | Deduct one point if the study was described as double blind but the method of blinding was inappropriate (e.g., comparison of tablet vs. injection with no double dummy). | 0 |

*Jadad scores range from 0 to 5. Trial scoring 3 or greater is considered as good quality trial (‘good trials’ = Jadad score of 4 or 5) vs. ‘poor trials’ = Jadad score of 1-3*

Jaded score for this study is 4/5, considering this is good quality trial.

### HIRULOG 1995

| Enrollment period | Mar 1993 - July 1994 | |
| --- | --- | --- |
| Study design | Multicenter, double blind, randomized trial | |
| Study aim | To studied whether these complications could be prevented when the direct thrombin inhibitor bivalirudin (Hirulog) was used in place of heparin. | |
| Outcome | Bivalirudin was at least as effective as high-dose heparin in preventing ischemic complications in patients who underwent angioplasty for unstable angina, and it carried a lower risk of bleeding. Bivalirudin, as compared with heparin, reduced the risk of immediate ischemic complications in patients with post infarction angina, but this difference was no longer apparent after six months | |
| Risk of bias | | |
| **Item** | **Author’s Judgement** | **Description** |
| Random sequence generation  (selection bias) | Unclear risk | Insufficient information to permit judgement of ‘Yes’ or ‘No’.  Comment: The allocation sequence generation was not defined clearly. |
| Allocation concealment (selection bias) | Unclear risk | Insufficient information to permit judgement of ‘Yes’ or ‘No’.  Comment: Method of concealment is not described to allow for definite judgement. |
| Blinding of participants and personnel (performance bias) | Unclear risk | Insufficient information to permit judgement of ‘Yes’ or ‘No’.  Comment: Blinding of participants and personnel were not defined clearly. |
| Blinding of outcome assessment  (detection bias) | Low risk | The outcome assessment was blinded.  Quote: “Every two months, an independent data safety and monitoring committee reviewed data on the incidence of major hemorrhage, myocardial infarction, and death and made recommendations to the steering committee about continuing the study”.  Comment: Probably done |
| Incomplete outcome data (attrition bias) | Low risk | Incomplete outcome data were adequately addressed.  Quote: “Base-line data were missing for 3 of the 4098 patients who underwent angioplasty (0.07 percent), angiographic data for 30 (0.7 percent), and follow-up data for 195 patients (4.8 percent)”  Comment: Probably done |
| Selective reporting  (reporting bias) | Low Risk | The reports of the study was free of suggestion of selective outcome reporting.  Comment: The study protocol is available and all of the study’s pre-specified (primary and secondary) outcomes that are of interest in the review have been reported in the pre-specified way. |
| Other bias | Low Risk | The study appears to be free of other sources of bias. |

**Jadad score**

| NO | ITEM | SCORE |
| --- | --- | --- |
| 1 | Was the study described as randomized (this includes words such as randomly, random, and randomization)? | 1 |
| 2 | Was the method used to generate the sequence of randomization described and appropriate (table of random numbers, computer generated, etc.? | 0 |
| 3 | Was the study described as double blind? | 1 |
| 4 | Was the method of double blinding described and appropriate (identical placebo, active placebo, dummy, etc.? | 0 |
| 5 | Was there a description of withdrawals and dropouts? | 1 |
| 6 | Deduct one point if the method used to generate the sequence of randomization was described and it was inappropriate (patients were allocated alternately, or according to date of birth, hospital number, etc.). | 0 |
| 7 | Deduct one point if the study was described as double blind but the method of blinding was inappropriate (e.g., comparison of tablet vs. injection with no double dummy). | 0 |

*Jadad scores range from 0 to 5. Trial scoring 3 or greater is considered as good quality trial (‘good trials’ = Jadad score of 4 or 5) vs. ‘poor trials’ = Jadad score of 1-3*

Jaded score for this study is 3/5, considering this is poor quality trials.

### HORIZON-AMI 2008

| Enrollment period | Mar 2005 - May 2007 | |
| --- | --- | --- |
| Study design | Multicenter, open-label , randomized trial | |
| Study aim | To evaluate safety and efficacy of bivalirudin in patients with ST-segment elevation myocardial infarction who presented within 12 hours after the onset of symptoms and who were undergoing primary PCI. | |
| Outcome | In patients with evolving ST-segment elevation myocardial infarction who are undergoing primary PCI, the use of bivalirudin alone, as compared with heparin plus a GPI, results in significantly reduced 30-day rates of major bleeding and increased event-free survival. | |
| **Risk of bias** | | |
| **Item** | **Author’s Judgement** | **Description** |
| Random sequence generation  (selection bias) | Low Risk | The allocation sequence generation was defined clearly.  Quote: "Randomization was performed with the use of a computerized, interactive voice response system and a dynamic (minimization) allocation scheme".  Comment: Probably done |
| Allocation concealment (selection bias) | Low Risk | Allocation was adequately concealed.  Quote: "Telephone randomization was performed"  Comment: Probably done |
| Blinding of participants and personnel (performance bias) | Low Risk | No blinding, but the review authors judge that the outcome and the outcome measurement are not likely to be influenced by lack of blinding.  Quote: "Patients were randomly assigned, in an open-label fashion" and "logistic complexities of the trial necessitated an open-label design, introducing potential bias. However, compliance with the protocol procedure and the study medications was high"  Comment: Probably done |
| Blinding of outcome assessment  (detection bias) | Low Risk | The outcome assessment was blinded.  Quote: "An independent clinical events committee that was unaware of the treatment assignments adjudicated all end-point events by reviewing the medical records."  Comment: Probably done |
| Incomplete outcome data (attrition bias) | Low Risk | Incomplete outcome data were adequately addressed.  Quote: “At 30 days: 23/1800 missing from intervention group (10 withdrew consent, 13 were lost to follow-up); 24/1802 missing from control group (9 withdrew consent, 15 were lost to follow-up)”  Comment: Probably done |
| Selective reporting  (reporting bias) | High Risk | The reports of the study was not entirely free of suggestion of selective outcome reporting.  Quote: "patients who were assigned to receive bivalirudin alone, as compared with those who were assigned to receive heparin plus a GPI, had a significantly reduced rate of net adverse clinical events (9.2% vs. 12.1%; relative risk, 0.76; 95% confidence interval [CI], 0.63 to 0.92; P = 0.005)"  Comment: On of the primary outcome, NACE was concluded to be significantly reduced in intervention group but the p value reported was equivalent to 0.05 |
| Other bias | Unclear Risk | Insufficient information to assess whether an important risk of bias exists  Comment: The study was partly sponsored by the company of the intervention drug. |

**Jadad score**

| NO | ITEM | SCORE |
| --- | --- | --- |
| 1 | Was the study described as randomized (this includes words such as randomly, random, and randomization)? | 1 |
| 2 | Was the method used to generate the sequence of randomization described and appropriate (table of random numbers, computer generated, etc.? | 1 |
| 3 | Was the study described as double blind? | 0 |
| 4 | Was the method of double blinding described and appropriate (identical placebo, active placebo, dummy, etc.? | 0 |
| 5 | Was there a description of withdrawals and dropouts? | 1 |
| 6 | Deduct one point if the method used to generate the sequence of randomization was described and it was inappropriate (patients were allocated alternately, or according to date of birth, hospital number, etc.). | 1 |
| 7 | Deduct one point if the study was described as double blind but the method of blinding was inappropriate (e.g., comparison of tablet vs. injection with no double dummy). | 0 |

*Jadad scores range from 0 to 5. Trial scoring 3 or greater is considered as good quality trial (‘good trials’ = Jadad score of 4 or 5) vs. ‘poor trials’ = Jadad score of 1-3*

Jaded score for this study is 4/5, considering this is good quality trial.

### ISAR-REACT 3 2010

| Enrollment period | Sept 2005-Jan 2008 | |
| --- | --- | --- |
| Study design | Multicenter, double blind, randomized trial | |
| Study aim | Whether bivalirudin is superior to unfractionated heparin in patients with stable or unstable angina who undergo percutaneous coronary intervention (PCI) after pretreatment with clopidogrel | |
| Outcome | In patients with stable and unstable angina who underwent PCI after pretreatment with clopidogrel, bivalirudin did not provide a net clinical benefit (i.e., it did not reduce the incidence of the composite end point of death, myocardial infarction, urgent target-vessel revascularization, or major bleeding) as compared with unfractionated heparin, but it did significantly reduce the incidence of major bleeding. | |
| Risk of bias | | |
| **Item** | **Author’s Judgement** | **Description** |
| Random sequence generation  (selection bias) | Unclear Risk | Insufficient information to permit judgement of ‘Yes’ or ‘No’.  Comment: The allocation sequence generation was not defined clearly. |
| Allocation concealment (selection bias) | Low Risk | Allocation was adequately concealed.  Quote: "use of opaque, sealed envelopes that contained the assignment"  Comment: Probably done |
| Blinding of participants and personnel (performance bias) | Low Risk | Blinding of participants and key study personnel ensured, and unlikely that the blinding could have been broken.  Quote: “Double-blinding was achieved by using identical vials for the study drugs in the two groups. Unblinding of the study groups was first done after completion of the statistical analyses”.  Comment: Probably done |
| Blinding of outcome assessment  (detection bias) | Low Risk | The outcome assessment was blinded.  Quote: “All events were adjudicated and classified by an event-adjudication committee whose members were unaware of the treatment assignments”.  Comment: Probably done |
| Incomplete outcome data (attrition bias) | Low Risk | Incomplete outcome data were adequately addressed.  Quote: “Only 1 patient withdrew from the study and before receiving treatment and was excluded from analysis”  Comment: Probably done |
| Selective reporting  (reporting bias) | Low Risk | The reports of the study was free of suggestion of selective outcome reporting.  Comment: The study protocol is available and all of the study’s pre-specified (primary and secondary) outcomes that are of interest in the review have been reported in the pre-specified way. |
| Other bias | Low Risk | The study appears to be free of other sources of bias. |

**Jadad score**

| NO | ITEM | SCORE |
| --- | --- | --- |
| 1 | Was the study described as randomized (this includes words such as randomly, random, and randomization)? | 1 |
| 2 | Was the method used to generate the sequence of randomization described and appropriate (table of random numbers, computer generated, etc.? | 0 |
| 3 | Was the study described as double blind? | 1 |
| 4 | Was the method of double blinding described and appropriate (identical placebo, active placebo, dummy, etc.? | 1 |
| 5 | Was there a description of withdrawals and dropouts? | 1 |
| 6 | Deduct one point if the method used to generate the sequence of randomization was described and it was inappropriate (patients were allocated alternately, or according to date of birth, hospital number, etc.). | 0 |
| 7 | Deduct one point if the study was described as double blind but the method of blinding was inappropriate (e.g., comparison of tablet vs. injection with no double dummy). | 1 |

*Jadad scores range from 0 to 5. Trial scoring 3 or greater is considered as good quality trial (‘good trials’ = Jadad score of 4 or 5) vs. ‘poor trials’ = Jadad score of 1-3*

Jaded score for this study is 5/5, considering this is good quality trial. The method to generate the sequence was unclear but the patients were randomly assigned in a double blind manner to receive either bivalirudin or unfractionated heparin using sealed opaque envelopes containing the block randomization sequence for each participating centre.

### ISAR-REACT 4 2011

| Enrollment period | Sept 2005– Jan 2008 | |
| --- | --- | --- |
| Study design | Multicenter, double blind, randomized trial | |
| Study aim | Compare combination of GPIs and heparin VERSUS bivalirudin in patients with non–ST-segment elevation myocardial infarction undergoing percutaneous coronary intervention (PCI). | |
| Outcome | Abciximab and unfractionated heparin, as compared with bivalirudin, failed to reduce the rate of the primary end point and increased the risk of bleeding among patients with non–ST-segment elevation myocardial infarction who were undergoing PCI. | |
| Risk of bias | | |
| **Item** | **Author’s Judgement** | **Description** |
| Random sequence generation  (selection bias) | Unclear risk | Insufficient information to permit judgement of ‘Yes’ or ‘No’.  Comment: The allocation sequence generation was not defined clearly. |
| Allocation concealment (selection bias) | Low risk | Allocation was adequately concealed.  Quote: "Assignments were made with the use of sealed opaque envelopes (concealed assignments)".  Comment: Probably done |
| Blinding of participants and personnel (performance bias) | Low Risk | Blinding of participants and key study personnel ensured, and unlikely that the blinding could have been broken.  Quote: “Patients at each participating center underwent randomization in a double-blind manner by means of a double-dummy-drug strategy”.  Comment: Probably done |
| Blinding of outcome assessment  (detection bias) | Low Risk | The outcome assessment was blinded.  Quote: “All events were adjudicated and classified by an event-adjudication committee whose members were unaware of the treatment assignments”.  Comment: Probably done |
| Incomplete outcome data (attrition bias) | Low Risk | Incomplete outcome data were adequately addressed.  Quote: “4 patients were excluded in the analysis because the operator decided not to perform planned intervention.(low risk lesion)”  Comment: Probably done |
| Selective reporting  (reporting bias) | Low Risk | The reports of the study was free of suggestion of selective outcome reporting.  Comment: The study protocol is available and all of the study’s pre-specified (primary and secondary) outcomes that are of interest in the review have been reported in the pre-specified way. |
| Other bias | Low Risk | The study appears to be free of other sources of bias. |

**Jadad score**

| NO | ITEM | SCORE |
| --- | --- | --- |
| 1 | Was the study described as randomized (this includes words such as randomly, random, and randomization)? | 1 |
| 2 | Was the method used to generate the sequence of randomization described and appropriate (table of random numbers, computer generated, etc.? | 0 |
| 3 | Was the study described as double blind? | 1 |
| 4 | Was the method of double blinding described and appropriate (identical placebo, active placebo, dummy, etc.? | 1 |
| 5 | Was there a description of withdrawals and dropouts? | 1 |
| 6 | Deduct one point if the method used to generate the sequence of randomization was described and it was inappropriate (patients were allocated alternately, or according to date of birth, hospital number, etc.). | 0 |
| 7 | Deduct one point if the study was described as double blind but the method of blinding was inappropriate (e.g., comparison of tablet vs. injection with no double dummy). | 1 |

*Jadad scores range from 0 to 5. Trial scoring 3 or greater is considered as good quality trial (‘good trials’ = Jadad score of 4 or 5) vs. ‘poor trials’ = Jadad score of 1-3*

Jaded score for this study is 5/5, considering this is good quality trial. The method of the generation sequence was unclear but the study is double blind and the method is appropriate whereby the patients at each participating center underwent randomization in a double-blind manner by means of a double-dummy-drug strategy.

### MATRIX 2015

| Enrollment period | Oct 2011 - Nov 2014 | |
| --- | --- | --- |
| Study design | Multicenter, open labelled, randomized trial | |
| Study aim | To assess whether bivalirudin is superior to unfractionated heparin and discretionary use of GPIs in patients with an acute coronary syndrome who were undergoing coronary angiography and anticipated percutaneous coronary intervention (PCI) with access through the radial or femoral route. | |
| Outcome | Among patients with acute coronary syndromes undergoing invasive treatment, neither the rate of major adverse cardiovascular events nor the rate of net adverse clinical events was significantly lower with bivalirudin than with unfractionated heparin and discretionary use of GPIs.  The post-PCI infusion of bivalirudin for at least 4 hours after the intervention did not result in a lower rate of the composite outcome of ischemic and bleeding events, including stent thrombosis, than the rate with no post-PCI infusion. | |
| **Item** | **Author’s Judgement** | **Description** |
| Random sequence generation  (selection bias) | Low Risk | The allocation sequence generation was defined clearly.  Quote: "Patients were randomly assigned, in a 1:1 ratio", "Patients who were assigned to the bivalirudin group were subsequently randomly assigned, in a 1:1 ratio" and "randomization sequences were computer generated, blocked, and stratified"  Comment: Probably done |
| Allocation concealment (selection bias) | Low Risk | Allocation was adequately concealed.  Quote: "Central randomization was concealed with the use of a Web-based system"  Comment: Probably done |
| Blinding of participants and personnel (performance bias) | High Risk | No blinding.  Quote: "All interventions were administered in an open label fashion"  Comment: No justification from author whether this will affect the outcome. |
| Blinding of outcome assessment  (detection bias) | Low Risk | The outcome assessment was blinded.  Quote: "An independent clinical-events committee whose members were unaware of study-group assignments adjudicated all suspected events"  Comment: Probably done |
| Incomplete outcome data (attrition bias) | Unclear Risk | Insufficient reporting of attrition/exclusions to permit judgement of ‘Yes’ or ‘No’.  Quote: “At 30 days: complete follow-up information was available for 7188 of 7213 patients (99.7%)”.  Comment: No reasons for missing data provided. |
| Selective reporting  (reporting bias) | Low Risk | The reports of the study was free of suggestion of selective outcome reporting.  Comment: The study protocol is available and all of the study’s pre-specified (primary and secondary) outcomes that are of interest in the review have been reported in the pre-specified way. |
| Other bias | Unclear Risk | Insufficient information to assess whether an important risk of bias exists.  Comment: The study was partly sponsored by the company of the intervention drug. |

**Jadad score**

| NO | ITEM | SCORE |
| --- | --- | --- |
| 1 | Was the study described as randomized (this includes words such as randomly, random, and randomization)? | 1 |
| 2 | Was the method used to generate the sequence of randomization described and appropriate (table of random numbers, computer generated, etc.? | 1 |
| 3 | Was the study described as double blind? | 0 |
| 4 | Was the method of double blinding described and appropriate (identical placebo, active placebo, dummy, etc.? | 0 |
| 5 | Was there a description of withdrawals and dropouts? | 1 |
| 6 | Deduct one point if the method used to generate the sequence of randomization was described and it was inappropriate (patients were allocated alternately, or according to date of birth, hospital number, etc.). | 0 |
| 7 | Deduct one point if the study was described as double blind but the method of blinding was inappropriate (e.g., comparison of tablet vs. injection with no double dummy). | 0 |

*Jadad scores range from 0 to 5. Trial scoring 3 or greater is considered as good quality trial (‘good trials’ = Jadad score of 4 or 5) vs. ‘poor trials’ = Jadad score of 1-3*

Jaded score for this study is 3/3, considering this is poor quality trial.

### NAPLES 2009

| Enrollment period | Oct 2005 - Feb 2008 | |
| --- | --- | --- |
| Study design | Single center, randomized trial | |
| Study aim | To evaluate the safety of bivalirudin monotherapy compared to UFH plus tirofiban in patients with DM undergoing elective PCI. | |
| Outcome | Because bivalirudin has proved its effectiveness in decreasing hemorrhagic events, its administration can be advocated in subjects deemed at  high risk. | |
| **Risk of bias** | | |
| **Item** | **Author’s Judgement** | **Description** |
| Random sequence generation  (selection bias) | Low Risk | The allocation sequence generation was defined clearly.  Quote: "The treatment assignment between the 2 groups was determined by a randomization block in a 1:1 ratio".  Comment: Probably done |
| Allocation concealment (selection bias) | Unclear Risk | Insufficient information to permit judgement of ‘Yes’ or ‘No’.  Comment: Method of concealment is not described to allow for definite judgement. |
| Blinding of participants and personnel (performance bias) | Unclear Risk | Insufficient information to permit judgement of ‘Yes’ or ‘No’.  Comment: Blinding of participants and personnel were not defined clearly. |
| Blinding of outcome assessment  (detection bias) | Low Risk | The outcome assessment was blinded.  Quote: "The end point classifications were determined by a clinical event committee unaware of the procedure details"  Comment: Probably done |
| Incomplete outcome data (attrition bias) | Low Risk | No missing outcome data was reported in the study.  Comment: Probably done |
| Selective reporting  (reporting bias) | Low Risk | The reports of the study was free of suggestion of selective outcome reporting.  Comment: The study protocol is available and all of the study’s pre-specified (primary and secondary) outcomes that are of interest in the review have been reported in the pre-specified way. |
| Other bias | Unclear Risk | Insufficient information to assess whether an important risk of bias exists.  Comment: No declaration made to as who sponsored the study. |

**Jadad score**

| NO | ITEM | SCORE |
| --- | --- | --- |
| 1 | Was the study described as randomized (this includes words such as randomly, random, and randomization)? | 1 |
| 2 | Was the method used to generate the sequence of randomization described and appropriate (table of random numbers, computer generated, etc.? | 1 |
| 3 | Was the study described as double blind? | 0 |
| 4 | Was the method of double blinding described and appropriate (identical placebo, active placebo, dummy, etc.? | 0 |
| 5 | Was there a description of withdrawals and dropouts? | 0 |
| 6 | Deduct one point if the method used to generate the sequence of randomization was described and it was inappropriate (patients were allocated alternately, or according to date of birth, hospital number, etc.). | 0 |
| 7 | Deduct one point if the study was described as double blind but the method of blinding was inappropriate (e.g., comparison of tablet vs. injection with no double dummy). | 0 |

*Jadad scores range from 0 to 5. Trial scoring 3 or greater is considered as good quality trial (‘good trials’ = Jadad score of 4 or 5) vs. ‘poor trials’ = Jadad score of 1-3*

Jaded score for this study is 2/5, considering this is poor quality trial.

### REPLACE-2 2003

| Enrollment period | Oct 2001 - Aug 2002 | |
| --- | --- | --- |
| Study design | Multicenter, double blind, randomized trial | |
| Study aim | To determine the efficacy of bivalirudin, with glycoprotein IIb/IIIa (Gp IIb/IIIa) inhibition on a provisional basis for complications during PCI, compared with heparin plus planned Gp IIb/IIIa blockade with regard to protection from periprocedural ischemic and hemorrhagic complications. | |
| Outcome | Bivalirudin with provisional Gp IIb/IIIa blockade is statistically not inferior to heparin plus planned Gp IIb/IIIa blockade during contemporary PCI with regard to suppression of acute ischemic end points and is associated with less bleeding | |
| Risk of bias | | |
| **Item** | **Author’s Judgement** | **Description** |
| Random sequence generation  (selection bias) | Unclear risk | Insufficient information to permit judgement of ‘Yes’ or ‘No’.  Comment: The allocation sequence generation was not defined clearly. |
| Allocation concealment (selection bias) | Low Risk | Allocation was adequately concealed.  Quote: "Patients were randomized in a double-blind fashion by a central telephone system"  Comment: Probably done |
| Blinding of participants and personnel (performance bias) | Low Risk | Blinding of participants and key study personnel ensured, and unlikely that the blinding could have been broken.  Quote: “Double blinding was maintained by using a double-dummy technique, where in hospital research pharmacists dispensed identical bivalirudin or heparin bolus syringes, bivalirudin or placebo infusion bags, and a GPI or placebo bolus and infusion”.  Comment: Probably done |
| Blinding of outcome assessment  (detection bias) | Low Risk | The outcome assessment was blinded.  Quote: “End point classification were made by a blinded clinical event committee”.  Comment: Probably done |
| Incomplete outcome data (attrition bias) | Low Risk | Incomplete outcome data were adequately addressed.  Quote: “At 30 day, data from 45 patients were excluded (8 withdrew consent; 16 lost to follow up; 21 incomplete data)”  Comment: Probably done |
| Selective reporting  (reporting bias) | Low Risk | The reports of the study was free of suggestion of selective outcome reporting.  Comment: The study protocol is available and all of the study’s pre-specified (primary and secondary) outcomes that are of interest in the review have been reported in the pre-specified way. |
| Other bias | Low Risk | The study appears to be free of other sources of bias. |

**Jadad score**

| NO | ITEM | SCORE |
| --- | --- | --- |
| 1 | Was the study described as randomized (this includes words such as randomly, random, and randomization)? | 1 |
| 2 | Was the method used to generate the sequence of randomization described and appropriate (table of random numbers, computer generated, etc.? | 0 |
| 3 | Was the study described as double blind? | 1 |
| 4 | Was the method of double blinding described and appropriate (identical placebo, active placebo, dummy, etc.? | 1 |
| 5 | Was there a description of withdrawals and dropouts? | 1 |
| 6 | Deduct one point if the method used to generate the sequence of randomization was described and it was inappropriate (patients were allocated alternately, or according to date of birth, hospital number, etc.). | 0 |
| 7 | Deduct one point if the study was described as double blind but the method of blinding was inappropriate (e.g., comparison of tablet vs. injection with no double dummy). | 0 |

*Jadad scores range from 0 to 5. Trial scoring 3 or greater is considered as good quality trial (‘good trials’ = Jadad score of 4 or 5) vs. ‘poor trials’ = Jadad score of 1-3*

Jaded score for this study is 4/5, considering this is good quality trial.
