# Supplementary material for: Safety and Effectiveness of Bivalirudin in Patients Undergoing Percutaneous Coronary Intervention: A Systematic Review and Meta-Analysis
Source: Front Pharmacol. 2017 Jul 11;8:410. doi: 10.3389/fphar.2017.00410 (PMC5504279; doi:10.3389/fphar.2017.00410)
Supplement: Supplementary file 3 [file DataSheet3.DOCX]

Supporting Information 3: Funnel plots

Figure 1: Funnel plot of comparison: bivalirudin vs heparin in angina and ACS, outcome: all-cause mortality at 30 days

Figure 2: Funnel plot of comparison: bivalirudin vs heparin in angina and ACS, outcome: myocardial infarction at 30 days

Figure 3: Funnel plot of comparison: bivalirudin vs heparin in angina and ACS, outcome: revascularisation at 30 days

Figure 4: Funnel plot of comparison: bivalirudin vs heparin in angina and ACS, outcome: stent thrombosis at 30 days

Figure 5: Funnel plot of comparison: bivalirudin vs heparin in angina and ACS, outcome: stroke at 30 days

Figure 6: Funnel plot of comparison: bivalirudin vs heparin in angina and ACS, outcome: major bleeding (study definition) at 30 days

Figure 7: Funnel plot of comparison: bivalirudin vs heparin in angina and ACS, outcome: major bleeding (TIMI definition) at 30 days
