# Supplementary material for: Safety and Effectiveness of Bivalirudin in Patients Undergoing Percutaneous Coronary Intervention: A Systematic Review and Meta-Analysis
Source: Front Pharmacol. 2017 Jul 11;8:410. doi: 10.3389/fphar.2017.00410 (PMC5504279; doi:10.3389/fphar.2017.00410)
Supplement: Supplementary file 4 [file DataSheet4.DOCX]

### Supporting information 4

### Meta-analysis for myocardial infarction outcome (HEAT-PPCI 2014 included)

Figure 1: Forest plot comparing 30-day myocardial infarction for patients randomized to bivalirudin versus heparin with provisional or routine GPI use

### (2) Meta-analysis for revascularisation outcome (HIRULOG 1995 included)

Figure 2: Forest plot comparing 30-day revascularisation for patients randomized to bivalirudin versus heparin with provisional or routine GPI use
