# Supplementary material for: Safety and Effectiveness of Bivalirudin in Patients Undergoing Percutaneous Coronary Intervention: A Systematic Review and Meta-Analysis
Source: Front Pharmacol. 2017 Jul 11;8:410. doi: 10.3389/fphar.2017.00410 (PMC5504279; doi:10.3389/fphar.2017.00410)
Supplement: Supplementary file 5 [file DataSheet5.DOCX]

### Supporting Information 5: Primary definition of bleeding used in different studies

| **Study/Acronym** | **Definition** |
| --- | --- |
| TIMI | Major bleeding was defined according to the Thrombolysis In Myocardial Infarction **(TIMI)** criteria:   - Any intracranial bleeding (excluding microhemorrhages <10 mm evident only on gradient-echo MRI) - Clinically overt signs of hemorrhage associated with a drop in hemoglobin of > 5 g/dL - Fatal bleeding (bleeding that directly results in death within 7 days) |
| ACUITY | The protocol definition of major bleeding was defined as :   - Intracranial or intraocular hemorrhage - Access-site hemorrhage requiring intervention - > 5-cm hematoma - Retroperitoneal - Reduction in hemoglobin concentration of > 4 g/dL without an overt source of bleeding - Reduction in hemoglobin concentration of > 3 g/dL with an overt source of bleeding - Reoperation for bleeding - Use of any blood product transfusion |
| REPLACE-2 2003 | Major bleeding was defined as:   - Intracranial, intraocular, or retroperitoneal - Overt blood loss with hemoglobin decrease > 3 g/dl - Any hemoglobin decrease > 4 g/dL - Transfusion of > 2 U blood products |
| BARC | Major bleeding was defined as Type 3–5 according to the Bleeding Academic Research Consortium (BARC) definition |
